# GLK-IKKβ signaling induces dimerization and translocation of the AhR-RORγt complex in IL-17A induction and autoimmune disease

**DOI:** 10.1126/sciadv.aat5401

**Published:** 2018-09-12

**Authors:** Huai-Chia Chuang, Ching-Yi Tsai, Chia-Hsin Hsueh, Tse-Hua Tan

**Affiliations:** 1 Immunology Research Center, National Health Research Institutes, Zhunan 35053, Taiwan.; 2 Department of Pathology and Immunology, Baylor College of Medicine, Houston, TX 77030, USA.

## Abstract

Retinoic-acid-receptor-related orphan nuclear receptor γt (RORγt) controls the transcription of interleukin-17A (IL-17A), which plays critical roles in the pathogenesis of autoimmune diseases. Severity of several human autoimmune diseases is correlated with frequencies of germinal center kinase–like kinase (GLK) (also known as MAP4K3)–overexpressing T cells; however, the mechanism of GLK overexpression–induced autoimmunity remains unclear. We report the signal transduction converging on aryl hydrocarbon receptor (AhR)–RORγt interaction to activate transcription of the IL-17A gene in T cells. T cell–specific GLK transgenic mice spontaneously developed autoimmune diseases with selective induction of IL-17A in T cells. In GLK transgenic T cells, protein kinase Cθ (PKCθ) phosphorylated AhR at Ser^36^ and induced AhR nuclear translocation. AhR also interacted with RORγt and transported RORγt into the nucleus. IKKβ (inhibitor of nuclear factor κB kinase β)–mediated RORγt Ser^489^ phosphorylation induced the AhR-RORγt interaction. T cell receptor (TCR) signaling also induced the novel RORγt phosphorylation and subsequent AhR-RORγt interaction. Collectively, TCR signaling or GLK overexpression induces IL-17A transcription through the IKKβ-mediated RORγt phosphorylation and the AhR-RORγt interaction in T cells. Our findings suggest that inhibitors of GLK or the AhR-RORγt complex could be used as IL-17A–blocking agents for IL-17A–mediated autoimmune diseases.

## Teaser

The kinase GLK induces dimerization of AhR-RORγt, leading to induction of the cytokine IL-17A and autoimmune responses.

## INTRODUCTION

Autoimmune diseases, which are chronic, debilitating, and life-threatening, arise from immune system overactivation. T helper 17 cells [T_H_17, interleukin-17A (IL-17A)–producing CD4^+^ T cells] and IL-17A play critical roles in the pathogenesis of autoimmune diseases (*1*, *2*). Induction of IL-17A occurs in the synovial fluids from rheumatoid arthritis (RA) patients (*3*) and in the renal biopsies from systemic lupus erythematosus (SLE) patients (*1*). IL-17A facilitates differentiation of osteoclasts, which lead to arthritis (*3*). IL-17A also promotes B cell proliferation and class switch recombination, contributing to autoantibody production and autoimmune responses (*4*). Furthermore, IL-17A overexpression causes tissue damage by inducing infiltration of neutrophils and macrophages through multiple chemokines (*1*, *2*, *5*). IL-17A knockout (KO) or IL-17A blockade abolishes disease development in animal autoimmune models such as experimental autoimmune encephalomyelitis (EAE) and collagen-induced arthritis (CIA) (*2*). Thus, understanding the mechanism(s) controlling IL-17A transcription may lead to the discovery of novel therapeutic targets for autoimmune diseases.

IL-1β, IL-6, IL-21, and IL-23 facilitate T_H_17 differentiation (*1*, *2*, *6*, *7*). The T_H_17 lineage–specific transcription factor RORγt binds to the IL-17A promoter and controls IL-17A gene transcription (*8*, *9*). Transforming growth factor–β (TGF-β)–induced Foxp3, a regulatory T cell (T_reg_) lineage–specific transcription factor, suppresses IL-17A expression by interacting with and inactivating RORγt (*10*, *11*). Besides RORγt, several non–lineage-specific transcription factors play important roles in regulating IL-17A transcription. IL-6– or IL-23–stimulated signal transducer and activator of transcription 3 (STAT3) binds to the IL-17A promoter and enhances IL-17A transcription (*12*, *13*). ROCK (Rho-associated, coiled-coil-containing protein kinase)–phosphorylated interferon (IFN) regulatory factor 4 (IRF4) also promotes IL-17A transcription upon TGF-β signaling (*14*). Basic leucine zipper ATF-like transcription factor (BATF) controls T_H_17 differentiation through binding to the intergenic elements between the IL-17A and IL-17F genes, as well as to the IL-21 or IL-22 promoters (*15*). Krüppel-like factor 4 (KLF4) binds to the IL-17A promoter and contributes to T_H_17 differentiation independent of RORγt expression (*16*). Aryl hydrocarbon receptor (AhR) promotes T_H_17 polarization by inducing IL-17A transcription (*17*, *18*) and inhibiting the negative regulator STAT1 during T_H_17 differentiation (*19*). Conversely, T cell–specific AhR KO mice have impaired T_H_17 differentiation and are resistant to T_H_17-mediated experimental autoimmune arthritis (*20*). Various pathogenic T_H_17 subpopulations can be derived in vitro under different conditions (*21*). Nevertheless, the in vivo roles of these distinct T_H_17 subpopulations in the pathogenesis of autoimmune diseases remain unclear.

MAP4K3 (also named GLK) is a mammalian Ste20-like serine/threonine kinase that was first identified as an upstream activator for the c-Jun N-terminal kinase pathway (*22*, *23*). GLK plays an important role in T cell receptor (TCR) signaling by directly phosphorylating and activating PKCθ, leading to activation of IKKβ [inhibitor of nuclear factor κB (NF-κB) kinase β] and NF-κB in T cells (*24*). Moreover, clinical samples from patients with autoimmune diseases, such as SLE, RA, or adult-onset Still’s disease, show markedly increased GLK expression in T cells; the frequencies of GLK-expressing T cells are positively correlated with disease severity (*24*–*26*). Consistent with the correlation of high levels of GLK expression with autoimmune disease, GLK-deficient mice are resistant to T_H_17-mediated EAE induction or CIA induction and display lower T_H_17 responses (*24*, *26*). Besides T_H_17, differentiation of T_H_1 and T_H_2 is also impaired in GLK-deficient T cells because of impaired TCR signaling (*24*). However, it remains unclear how GLK overexpression contributes to multiple human autoimmune diseases. To understand the pathogenic mechanism of GLK overexpression–induced autoimmune diseases, we generated and characterized T cell–specific GLK transgenic (Lck-GLK Tg) mice. Here, we report that GLK signaling induces a novel interaction between AhR and phospho-RORγt through PKCθ and IKKβ, leading to IL-17A transcriptional activation and autoimmune responses.

## RESULTS

### Lck-GLK Tg mice develop autoimmune diseases through IL-17A

**Fig. 1:**
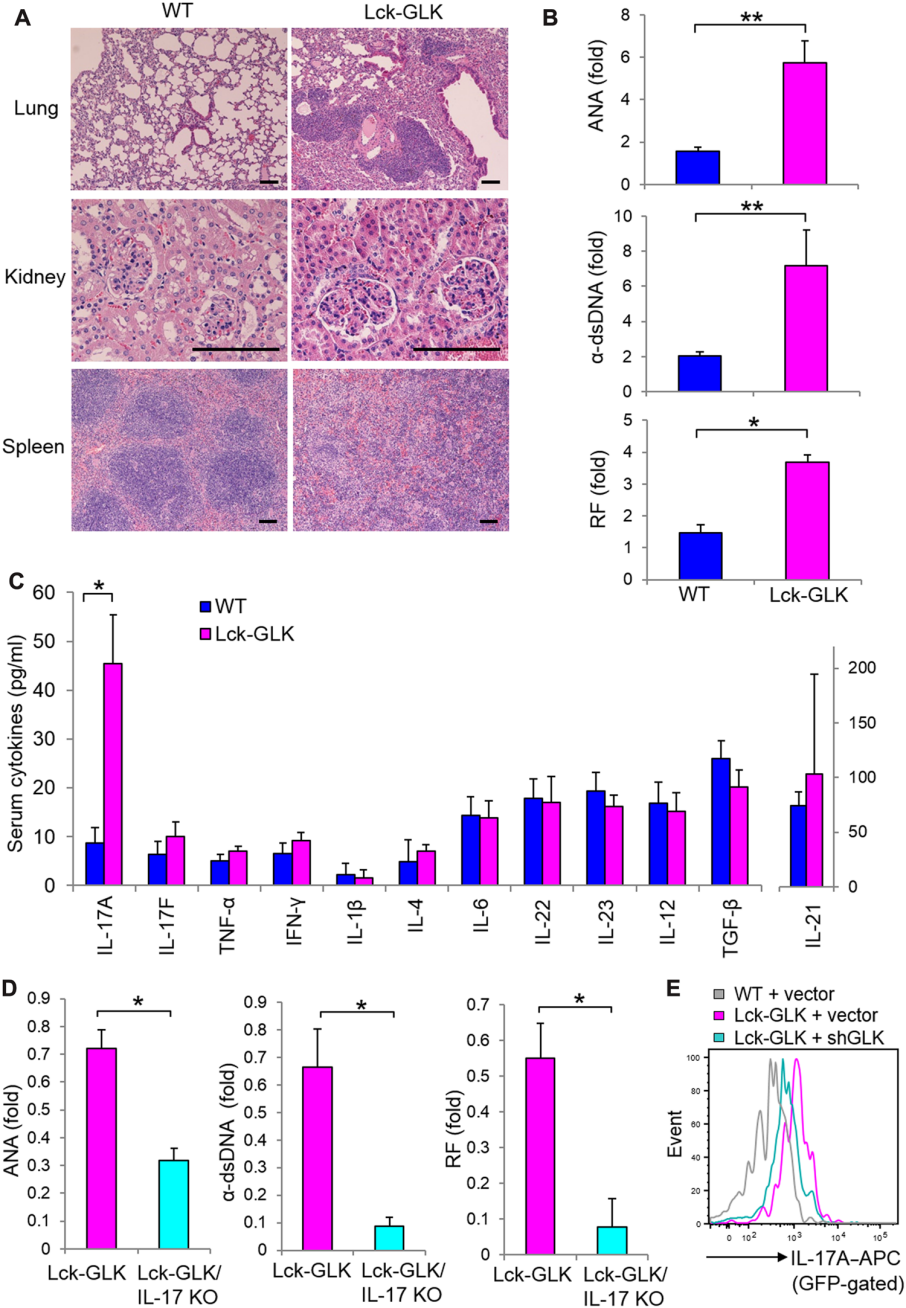
Lck-GLK Tg mice display autoimmune phenotypes and selectively increased serum IL-17A levels. (**A**) Hematoxylin and eosin (H&E)–stained sections of the indicated organs from 16-week-old mice. Scale bars, 100 μm. (**B**) Levels of serum autoantibodies from 20-week-old mice were determined by ELISAs. The levels are presented relative to the value from one of the wild-type (WT) mice. WT, *n* = 7; Lck-GLK, *n* = 8. (**C**) The serum levels of cytokines in 4-week-old mice were determined by ELISAs. WT, *n* = 20; Lck-GLK, *n* = 16. (**D**) The serum levels of autoantibodies in 20-week-old Lck-GLK and Lck-GLK/IL-17A KO mice were determined by ELISAs. The levels are presented relative to the value from one of the Lck-GLK mice. *n* = 6 per group. (**E**) IL-17A expression was attenuated by GLK shRNA. Murine primary splenic T cells were transfected with green fluorescent protein (GFP)–human GLK shRNA and a control GFP vector. The transfected T cells were stimulated with anti-mouse CD3 antibodies for 3 hours and then determined by flow cytometry at day 3 after transfection. Data show the events of IL-17A–producing T cells (GFP-gated). WT, wild-type littermate controls; Lck-GLK, T cell–specific GLK Tg mice; Lck-GLK/IL-17A KO, Lck-GLK;IL-17A–deficient mice; ANA, antinuclear antibody; α–double-stranded DNA (dsDNA), anti-dsDNA antibody; RF, rheumatoid factor; APC, allophycocyanin. Data shown are representative of three independent experiments. *P < 0.05, **P < 0.01 (two-tailed Student’s *t* test).

To study the consequence of GLK overexpression in vivo, we generated Lck-GLK Tg mice (fig. S1, A and B). Lck-GLK Tg mice displayed normal development of T cells and B cells (fig. S1, C to H); however, the mice displayed paralysis of the hindlimb and tail, clouding of the eye, or symptoms of proctitis and dermatitis between 8 and 16 weeks of age (fig. S2A). Lck-GLK Tg mice also showed hepatosplenomegaly and enlargement of lymph nodes and kidneys (fig. S2B). Histology staining showed the induction of pneumonia, nephritis, and spleen abnormality in these mice (Fig. 1A). Induction of serum autoantibodies in Lck-GLK Tg mice (Fig. 1B) also suggests the development of autoimmune responses in these mice. To study which proinflammatory cytokines contribute to autoimmune diseases in Lck-GLK Tg mice, we determined serum cytokines via enzyme-linked immunosorbent assay (ELISA). Surprisingly, only IL-17A was selectively induced in the sera of 4-week-old Lck-GLK Tg mice (Fig. 1C), whereas the proinflammatory cytokines IFN-γ and tumor necrosis factor–α (TNF-α) were not induced. Consistently, in vitro T_H_17 differentiation of Lck-GLK Tg T cells was increased compared to that of wild-type T cells (fig. S2C), whereas in vitro T_H_1 differentiation of Lck-GLK Tg T cells was unaffected (fig. S2C). Moreover, IL-17A production was induced in unstimulated T cells of Lck-GLK Tg mice compared to that of wild-type mice (fig. S2D). To rule out the potential position effect of the transgene, we also characterized the second Lck-GLK Tg mouse line (Lck-GLK #2) (fig. S1B). The second line of Lck-GLK Tg mice also displayed GLK overexpression in T cells (fig. S1B) and induction of serum IL-17A levels (fig. S2E). These data suggest that GLK overexpression in T cells induces IL-17A production in mice.

To demonstrate the pathogenic role of IL-17A in Lck-GLK Tg mice, we bred Lck-GLK Tg mice with IL-17A–deficient mice. GLK-induced serum IL-17A levels were significantly decreased by IL-17A deficiency, while other inflammatory cytokine levels were unaffected (fig. S3A). Moreover, autoantibody levels were also significantly reduced in Lck-GLK Tg/IL-17A–deficient mice compared to those in Lck-GLK Tg mice (Fig. 1D). Lck-GLK Tg/IL-17A–deficient mice displayed a reduction of infiltrating inflammatory cells in the kidneys, the liver, and the lung, while showing normal distribution of white pulp and red pulp in the spleen, compared to those in Lck-GLK Tg mice (fig. S3B). The data suggest that IL-17A contributes to autoimmune responses in Lck-GLK Tg mice. To further demonstrate that the induction of IL-17A is due to GLK overexpression, we treated Lck-GLK T cells with GLK short hairpin RNA (shRNA). IL-17A overproduction was abolished by GLK shRNA knockdown in T cells purified from Lck-GLK Tg mice (Fig. 1E). These results demonstrate that GLK overexpression induces IL-17A overproduction and subsequent autoimmune phenotypes in mice.

### GLK induces IL-17A transcription by activating AhR and RORγt

**Fig. 2:**
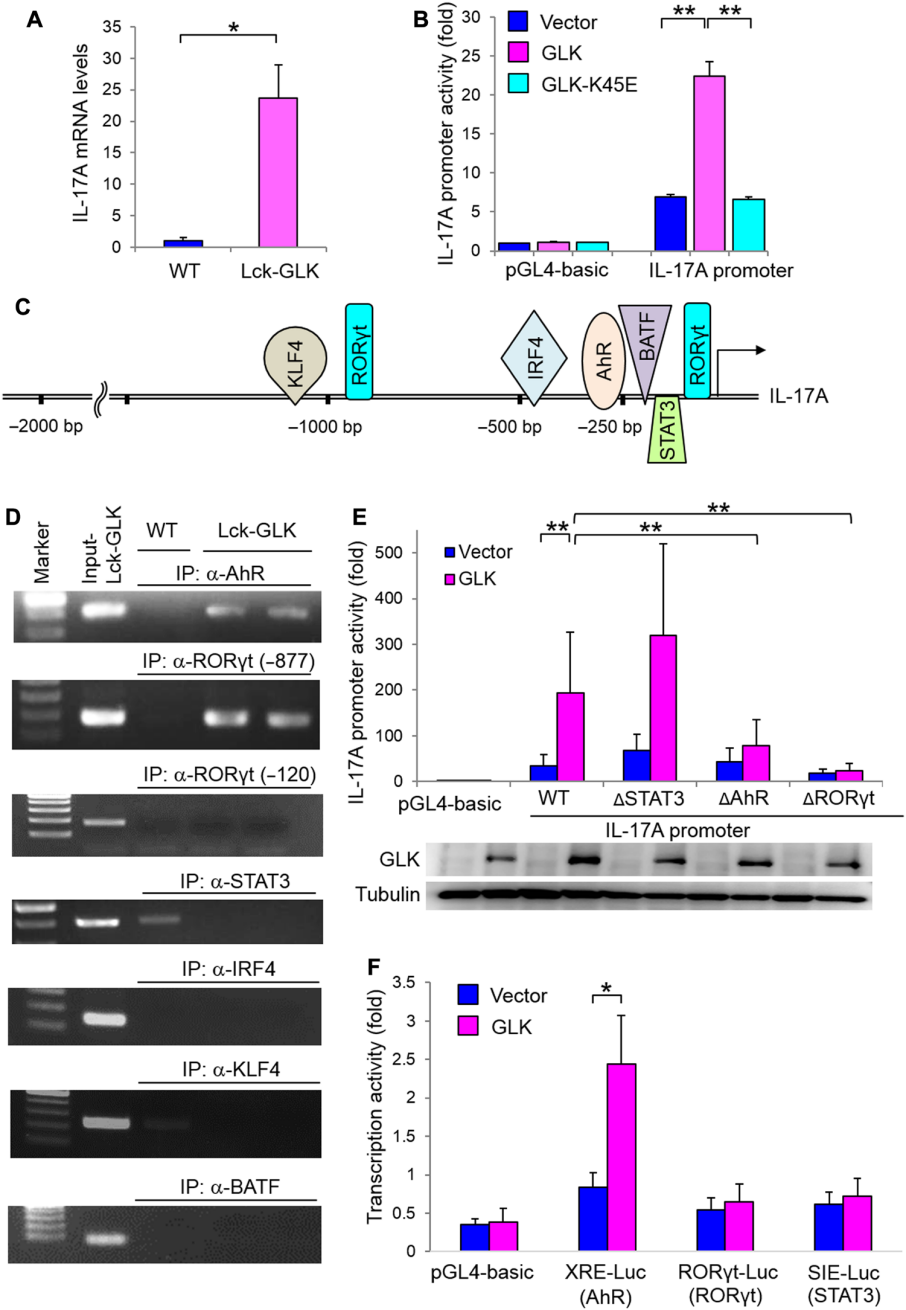
GLK enhances IL-17A expression by inducing AhR and RORγt. (**A**) Murine IL-17A mRNA levels in peripheral blood T cells from mice were analyzed by real-time polymerase chain reaction (PCR). The expression levels of IL-17A were normalized to Mrpl32 levels. The fold changes are presented relative to the value of WT mice. Means ± SEM are shown. *n* = 4 per group. (**B**) Luciferase reporter activity of the IL-17A promoter. Jurkat T cells were cotransfected with the plasmid encoding GLK or GLK kinase-dead (GLK-K45E) mutant plus the IL-17A promoter (2 kb) construct. Means ± SEM are shown. (**C**) Schematic diagram of transcription factors on the IL-17A promoter. bp, base pair. (**D**) The binding of AhR, RORγt, STAT3, IRF4, KLF4, or BATF to the IL-17A promoter in T cells from mice was analyzed by chromatin immunoprecipitation (IP) (ChIP)–PCR using immunocomplexes from individual IP experiments. (**E**) Luciferase reporter activity of the IL-17A mutant promoters. Jurkat T cells were cotransfected with empty vector or GLK plasmid plus the IL-17A promoter construct containing a mutated binding element for AhR, RORγt (−877), or STAT3. (**F**) Luciferase reporter activity of AhR, RORγt (−877), and STAT3 response element (XRE-Luc, RORγt-Luc, and SIE-Luc) in Jurkat T cells cotransfected with empty vector or plasmid encoding GLK. XRE, xenobiotic response element; SIE, sis-inducible element. WT, wild-type littermate controls; Lck-GLK, T cell–specific GLK Tg mice. Data shown are representative of three independent experiments. Means ± SEM of three independent experiments are shown (B, E, and F). *P < 0.05, **P < 0.01 (two-tailed Student’s *t* test).

Next, we studied the mechanism of GLK-induced IL-17A in T cells. The levels of IL-23 receptor and phosphorylated STAT3 were not increased in T cells of Lck-GLK Tg mice (fig. S4, A and B), suggesting that IL-17A overexpression is not due to enhancement of IL-23 signaling or IL-6/STAT3 signaling. Consistent with the IL-17A protein levels, mRNA levels of IL-17A were significantly increased in the purified T cells of Lck-GLK Tg mice compared to those of wild-type mice (Fig. 2A). We studied whether IL-17A overexpression is due to transcriptional activation of the IL-17A promoter. IL-17A promoter activities in Jurkat T cells were enhanced by GLK overexpression but not by GLK kinase-dead (K45E) mutant (Fig. 2B). Next, we studied the bindings of individual IL-17A transcription factors to the IL-17A promoter (Fig. 2, C and D). ChIP analyses showed that bindings of AhR and RORγt (−877) to the IL-17A promoter were induced in T cells of Lck-GLK Tg mice (Fig. 2D), whereas bindings of STAT3, IRF4, KLF4, and BATF to the IL-17A promoter were not enhanced (Fig. 2D). The binding of RORγt to the −120 region of the IL-17A promoter was not significantly induced (Fig. 2D); others reported similar findings (*27*, *28*). Consistent with ChIP data, mutation of the AhR-binding element or the RORγt-binding site (−877) abolished the GLK-enhanced IL-17A reporter activity, whereas mutation of the STAT3-binding site (Fig. 2E) or the RORγt-binding site (−120) did not affect the GLK-induced IL-17A reporter activity (fig. S4C). Notably, GLK overexpression induced AhR response element–driven reporter (XRE-Luc) activity (Fig. 2F), whereas RORγt (−877) or STAT3 response element–driven reporter (RORγt-Luc or SIE-Luc) activity was unaffected (Fig. 2F). These results suggest that GLK signaling induces IL-17A transcription by activating AhR and maybe RORγt.

### GLK controls IL-17A production and autoimmune responses through AhR

**Fig. 3:**
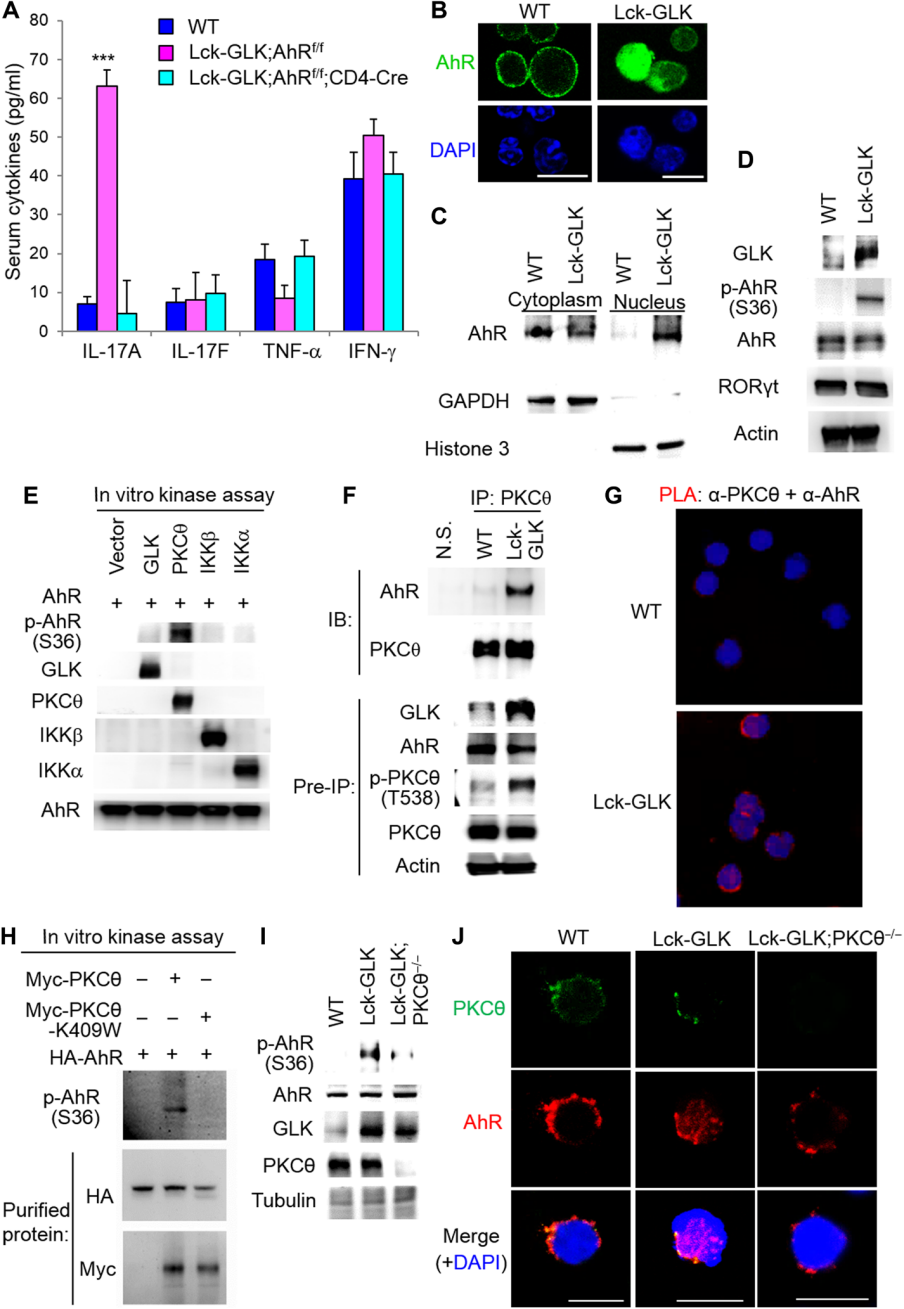
PKCθ phosphorylates AhR and induces its nuclear translocation. (**A**) The serum levels of cytokines from 4-week-old WT, Lck-GLK Tg, and Lck-GLK Tg/AhR cKO mice were determined by ELISAs. *n* = 8 per group. Means ± SEM are shown. WT, wild-type littermate controls; Lck-GLK, T cell–specific GLK Tg mice; Lck-GLK;AhR^f/f^;CD4-Cre, T cell–specific GLK Tg mice bred with AhR cKO mice. ***P < 0.001 (two-tailed Student’s *t* test). (**B**) Confocal microscopy analysis of subcellular localization of AhR in murine splenic T cells without stimulation. An anti-AhR antibody (clone RPT9, Abcam) was used. Original magnification, ×630; scale bars, 10 μm. (**C**) Immunoblotting analyses of AhR, glyceraldehyde-3-phosphate dehydrogenase (GAPDH), and histone 3 in cytoplasmic and nuclear fractions of primary splenic T cells from WT and Lck-GLK Tg mice. (**D**) Immunoblotting analysis of p-AhR (Ser^36^), AhR, and GLK in primary splenic T cells of WT and Lck-GLK Tg mice. (**E**) Immunoblotting analysis of AhR phosphorylation and indicated kinases in in vitro kinase assays using Flag-GLK, Flag-PKCθ, Flag-IKKβ, Flag-IKKα, and hemagglutinin (HA)–AhR (as the substrate) isolated from individually transfected HEK293T cells. (**F**) Coimmunoprecipitation of endogenous AhR with PKCθ from lysates of primary splenic T cells from WT and Lck-GLK mice without stimulation. N.S., normal serum. (**G**) Proximity ligation assays (PLAs) of interaction between endogenous PKCθ and AhR in peripheral blood T cells from WT or Lck-GLK Tg mice. Each red dot represents a direct interaction. T cell nucleus was stained with 4′,6-diamidino-2-phenylindole (DAPI) (blue). Images were captured with ×400 original magnification by a Leica DM2500 fluorescence microscope. (**H**) In vitro kinase assays of purified HA-tagged AhR plus either Myc-tagged PKCθ WT or PKCθ kinase-dead (K409W) mutant proteins. (**I**) Immunoblotting analysis of phosphorylated AhR (Ser^36^), AhR, GLK, and PKCθ in primary splenic T cells of WT, Lck-GLK Tg, and Lck-GLK Tg mice bred with PKCθ KO mice. (**J**) Confocal microscopy analysis of subcellular localization of AhR and PKCθ in primary splenic T cells of indicated mice. Original magnification, ×630; scale bars, 10 μm. WT, wild-type littermate controls; Lck-GLK, T cell–specific GLK Tg mice; Lck-GLK;PKCθ^−/−^, Lck-GLK Tg mice bred with PKCθ KO mice. Data shown are representative of three independent experiments.

To further demonstrate the role of AhR in promoting IL-17A production in Lck-GLK Tg mice, we bred Lck-GLK Tg mice with T cell–specific AhR conditional KO (AhR cKO: AhR^f/f^;CD4-Cre) mice. Serum IL-17A levels were drastically reduced in Lck-GLK/AhR cKO (Lck-GLK;AhR^f/f^;CD4-Cre) mice, whereas serum TNF-α and IFN-γ levels were unaffected by AhR deficiency (Fig. 3A). Levels of ANA, anti-dsDNA antibody, and RF were also decreased in Lck-GLK/AhR cKO mice compared to those in Lck-GLK Tg mice (fig. S5A). Histology staining showed that AhR KO abolished induction of nephritis and spleen abnormality in Lck-GLK Tg mice (fig. S5B). AhR KO also suppressed infiltration of inflammatory immune cells in the liver of Lck-GLK Tg mice (fig. S5B). The data indicate that AhR plays a critical role in the IL-17A overproduction and autoimmune responses in Lck-GLK Tg mice.

### PKCθ phosphorylates AhR at Ser^36^ and induces AhR nuclear translocation

Next, we studied the mechanism of GLK-induced AhR binding to the IL-17A promoter. The confocal images (using two different anti-AhR antibodies; Fig. 3B and fig. S5C) and subcellular fractionation experiments (Fig. 3C) showed that AhR nuclear translocation was enhanced in T cells of Lck-GLK Tg mice. In addition, we examined whether GLK signaling induces AhR nuclear translocation by enhancing phosphorylation of AhR. There is only one commercial anti–phospho-AhR antibody that detects phospho-Ser^36^ AhR; however, the role of Ser^36^ phosphorylation in AhR nuclear translocation has not been demonstrated (*29*). Immunoblotting analyses using the anti–phospho-AhR antibody for AhR phosphorylation showed that AhR Ser^36^ phosphorylation was enhanced in T cells of Lck-GLK Tg mice, as well as in anti-CD3–stimulated T cells (Fig. 3D and fig. S5D). These data suggest that GLK overexpression (and TCR signaling) may induce AhR activity by enhancing AhR Ser^36^ phosphorylation and nuclear translocation in T cells.

Next, we investigated which kinase is responsible for phosphorylation and nuclear translocation of AhR in T cells of GLK Tg mice. SGK1 (serum/glucocorticoid-regulated kinase 1) can stabilize T_H_17 population (*30*). The basal or TCR-induced SGK1 activation was unchanged in Lck-GLK T cells (fig. S5D), suggesting that SGK1 is not involved in GLK-induced AhR phosphorylation. GLK signaling in T cells induces kinase activities of PKCθ, IKKα, and IKKβ (*24*). To determine which kinase phosphorylates AhR, we immunoprecipitated GLK, PKCθ, IKKα, IKKβ, and AhR each from individually transfected human embryonic kidney (HEK) 293T cells and subjected them to in vitro kinase assays. The data showed that AhR Ser^36^ phosphorylation was drastically induced by PKCθ in vitro (Fig. 3E). We confirmed the specificity of the antibody for AhR Ser^36^ phosphorylation using a wild-type AhR or an S36A mutant transfectant by immunoblotting (fig. S5E). Immunofluorescence and confocal imaging analyses showed that PKCθ overexpression enhanced AhR nuclear translocation in Jurkat T cells (fig. S5F) and HEK293T cells (fig. S5G), whereas AhR-S36A mutation stayed in the cytoplasm even in PKCθ-overexpressing cells (fig. S5, F and G). The data indicate that PKCθ-mediated Ser^36^ phosphorylation of AhR stimulates AhR nuclear translocation. The interaction between PKCθ and AhR was induced in purified T cells of Lck-GLK Tg mice (Fig. 3F). Moreover, the protein-protein interaction/ALPHA (amplified luminescent proximity homogeneous assay) technology assays showed an interaction (<200 nm) between PKCθ and AhR, but not between GLK and AhR (fig. S6A). Furthermore, the fluorescence resonance energy transfer (FRET) analysis showed a direct interaction (1 to 10 nm) between PKCθ and AhR in PKCθ/AhR-cotransfected Jurkat T cells (fig. S6B). To study the subcellular localization and protein interaction (<40 nm) in vivo, we performed in situ PLA using probes corresponding to PKCθ and AhR. The PLA data showed strong signals in the cytoplasm of T cells from Lck-GLK Tg mice (Fig. 3G and fig. S6C). In situ PLA using probes corresponding to Myc and Flag tags showed similar results in Myc-PKCθ– and Flag-AhR–overexpressing HEK293T cells (fig. S6D). In vitro binding assays with purified PKCθ and AhR proteins further confirmed this direct interaction (fig. S6E). Furthermore, purified PKCθ phosphorylated AhR at Ser^36^, whereas purified kinase-dead (K409W) mutant of PKCθ did not (Fig. 3H). These data demonstrate that PKCθ directly interacts with and phosphorylates AhR at Ser^36^, leading to AhR nuclear translocation.

To verify the role of PKCθ in AhR nuclear translocation and its in vivo function, we generated PKCθ KO mice using transcription activator-like effector nuclease (TALEN) technology (fig. S7, A to C). We then bred Lck-GLK Tg mice with PKCθ KO mice to generate Lck-GLK;PKCθ^−/−^ mice. As expected, PKCθ KO abolished GLK-induced AhR Ser^36^ phosphorylation in T cells (Fig. 3I). Immunofluorescence and confocal imaging analyses showed that AhR was detected abundantly in the nucleus of T cells from Lck-GLK Tg mice (Fig. 3J). In contrast, AhR expression was detected in the cytoplasm, but not in the nucleus, of T cells from wild-type and Lck-GLK Tg/PKCθ KO (Lck-GLK;PKCθ^−/−^) mice (Fig. 3J). The serum levels of IL-17A and autoantibodies were significantly decreased in Lck-GLK;PKCθ^−/−^ mice compared to those in Lck-GLK Tg mice (fig. S7, D and E). Moreover, the inflammatory phenotypes were abolished in Lck-GLK Tg/PKCθ KO mice (fig. S7F). Together, these results indicate that GLK induces IL-17A production by activating PKCθ-AhR signaling in T cells.

### AhR interacts with RORγt and transports RORγt into the nucleus

**Fig. 4:**
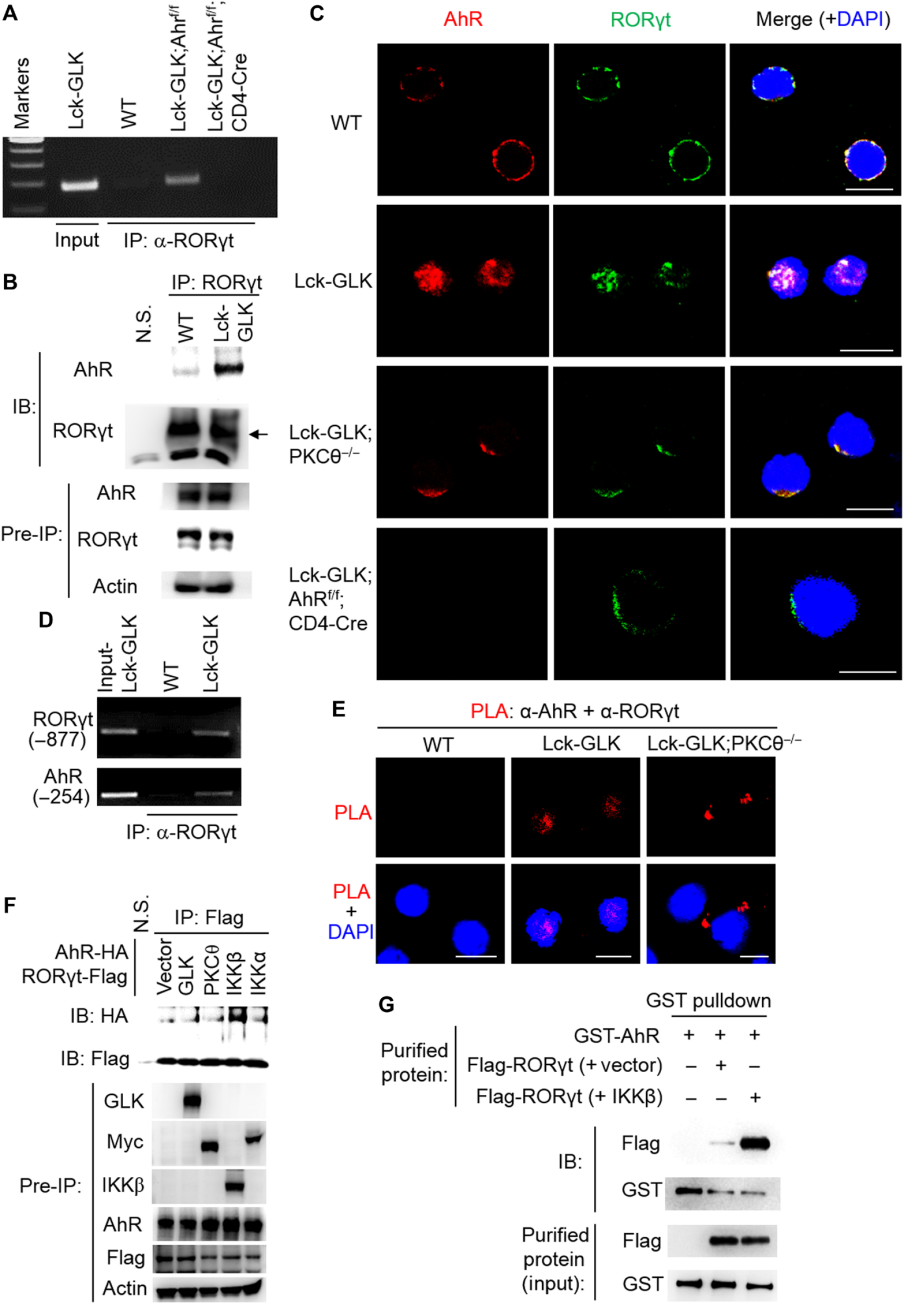
GLK induces RORγt binding to the IL-17A promoter through AhR and RORγt interaction. (**A**) Binding of RORγt to the IL-17A promoter in T cells from indicated mice was analyzed by ChIP-PCR. (**B**) Coimmunoprecipitation of endogenous AhR with RORγt using lysates of primary splenic T cells from WT and Lck-GLK mice without any stimulation. IB, immunoblotting. (**C**) Confocal microscopy analysis of subcellular localization of AhR and RORγt in primary T cells of WT, Lck-GLK Tg, Lck-GLK;PKCθ^−/−^, and Lck-GLK;AhR^f/f^;CD4-Cre mice. Original magnification, ×630; scale bars, 10 μm. (**D**) Binding of AhR and RORγt to the IL-17A promoter in T cells from mice was analyzed by ChIP-PCR using anti-RORγt immunocomplexes. (**E**) Confocal microscopy analysis of PLAs for the interaction between endogenous AhR and RORγt in peripheral blood T cells from WT, Lck-GLK Tg, and Lck-GLK;PKCθ^−/−^ mice. Original magnification, ×630; scale bars, 10 μm. (**F**) Coimmunoprecipitation experiments of HA-tagged AhR and Flag-tagged RORγt using lysates of HEK293T cells cotransfected with GLK–cyan fluorescent protein (CFP), PKCθ-Myc, IKKβ-CFP, or IKKα-Myc plasmid. (**G**) GST pulldown assays of purified Flag-tagged RORγt and GST-tagged AhR proteins. Flag-tagged RORγt proteins were eluted with Flag peptides using lysates of HEK293T cells cotransfected with Flag-RORγt plus either CFP-IKKβ or vector. For PLA, each red dot represents a direct interaction. T cell nucleus was stained with DAPI (blue). Data shown are representative of three independent experiments.

Paradoxically, both AhR- and RORγt-binding elements are required for the GLK-induced IL-17A reporter activity (Fig. 2E); however, GLK induced the activity of AhR, but not RORγt, response element (Fig. 2F). We suspected that AhR may facilitate induction of RORγt activity. We first studied whether GLK induces RORγt binding to the IL-17A promoter through AhR. ChIP data showed that the GLK-induced RORγt binding to the IL-17A promoter was abolished in AhR KO T cells (Fig. 4A). The data suggest that AhR facilitates the binding of RORγt to the IL-17A promoter. Next, we studied whether AhR interacts with RORγt. The interaction between endogenous AhR and RORγt was drastically enhanced in T cells of Lck-GLK Tg mice (Fig. 4B). Confocal imaging analysis showed colocalization of AhR and RORγt in the nucleus of T cells from Lck-GLK Tg mice (Fig. 4C). ChIP analysis using an anti-RORγt antibody also showed the binding of RORγt to the AhR-binding element of the IL-17A promoter in T cells of Lck-GLK mice (Fig. 4D), suggesting that RORγt binds to the AhR-binding element through the AhR-RORγt complex formation. Moreover, in situ PLA with probes corresponding to AhR and RORγt showed strong interaction signals in the nucleus of T cells from Lck-GLK Tg mice compared to those from wild-type mice (Fig. 4E). These data indicate direct interaction between AhR and RORγt in the nucleus of T cells from Lck-GLK Tg mice. In addition, similar to AhR nuclear translocation, GLK-induced RORγt nuclear translocation was abolished by PKCθ KO (Fig. 4C). The data support the idea that PKCθ-phosphorylated AhR recruits RORγt into the nucleus. AhR-mediated RORγt nuclear translocation is further confirmed by AhR KO; RORγt localized exclusively in the cytoplasm in T cells of Lck-GLK;AhR^f/f^;CD4-Cre mice even in the presence of functional PKCθ (Fig. 4C, bottom). Thus, this result rules out the possibility that PKCθ directly regulates RORγt nuclear translocation. Collectively, these results indicate that AhR directly interacts with and transports RORγt into the nucleus in GLK-overexpressing T cells.

### IKKβ-mediated RORγt Ser^489^ phosphorylation induces AhR-RORγt interaction

**Fig. 5:**
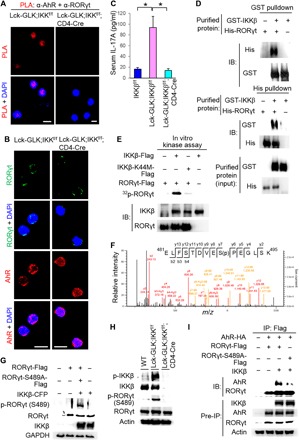
IKKβ phosphorylates RORγt Ser^489^, leading to RORγt binding to AhR. (**A**) Confocal microscopy analysis of PLAs for the interaction between endogenous AhR and RORγt in peripheral blood T cells from WT, Lck-GLK Tg, and Lck-GLK;IKKβ^f/f^;CD4-Cre mice. (**B**) Confocal microscopy analysis of subcellular localization of AhR and RORγt in primary splenic T cells of WT, Lck-GLK Tg, and Lck-GLK;IKKβ^f/f^;CD4-Cre mice. Original magnification, ×630; scale bars, 10 μm. (**C**) The serum levels of cytokines in 8-week-old mice were determined by ELISAs. IKKβ^f/f^, *n* = 6; Lck-GLK, *n* = 6; Lck-GLK; IKKβ^f/f^, *n* = 5. Means ± SEM are shown. *P < 0.05 (two-tailed Student’s *t* test). (**D**) Direct interaction between recombinant proteins of RORγt and IKKβ. GST or His pulldown assays of purified His-tagged RORγt and GST-tagged IKKβ proteins. (**E**) In vitro kinase assays of immunoprecipitated Flag-tagged RORγt and either IKKβ or IKKβ kinase-dead (K44M) mutant proteins from individual HEK293T transfectants. (**F**) Tandem MS (MS/MS) fragmentation spectra of the tryptic peptides of RORγt contain the phosphorylation of Ser^489^. *m/z*, mass/charge ratio. (**G**) Antibody specificity of anti–phospho-RORγt (Ser^489^) was demonstrated by immunoblotting using HEK293T cells cotransfected with CFP-tagged IKKβ plus either Flag-tagged RORγt WT or RORγt-S489A mutant. (**H**) Immunoblotting analyses of p-RORγt (Ser^489^), RORγt, p-IKKβ (Ser^180/181^), and IKKβ in primary splenic T cells of WT, Lck-GLK Tg, and Lck-GLK;IKKβ^f/f^; CD4-Cre mice. (**I**) Coimmunoprecipitation experiments of HA-tagged AhR and either Flag-tagged RORγt WT or RORγt-S489A mutant using lysates of HEK293T cells cotransfected with vector or IKKβ-CFP plasmid. WT, wild-type littermate controls; Lck-GLK, T cell–specific GLK Tg mice; Lck-GLK;IKKβ^f/f^;CD4-Cre, T cell–specific GLK Tg mice bred with IKKβ cKO mice. For PLA, each red dot represents a direct interaction. T cell nucleus was stained with DAPI (blue). Data shown (A, B, D, E, and G to I) are representative of three independent experiments.

Surprisingly, in situ PLA showed an interaction between AhR and RORγt even in the cytoplasm of Lck-GLK;PKCθ^−/−^ T cells (Fig. 4E). This result suggests that the interaction between AhR and RORγt is not regulated by AhR phosphorylation. Interaction between AhR and RORγt is still detectable in Lck-GLK;PKCθ^−/−^ T cells (Fig. 4E); this result may be due to a compensatory signaling event in PKCθ KO mice. To identify the kinase that stimulates the interaction between AhR and RORγt, we initially tested the potential role of GLK, IKKα, or IKKβ in the induction of AhR-RORγt interaction. We cotransfected the kinase GLK, PKCθ, IKKα, or IKKβ with AhR plus RORγt into HEK293T cells, followed by coimmunoprecipitation assays. The data showed that IKKβ overexpression enhanced the interaction between AhR and RORγt, whereas overexpression of GLK, PKCθ, and IKKα did not (Fig. 4F). Next, we tested whether IKKβ stimulates RORγt phosphorylation, which then induces the interaction of RORγt with AhR. Flag-tagged RORγt was purified from HEK293T cells cotransfected with RORγt plus either IKKβ or vector. Glutathione *S*-transferase (GST) pulldown assays showed that purified GST-tagged AhR recombinant proteins strongly interacted with the purified RORγt proteins from RORγt plus IKKβ-cotransfected cells (Fig. 4G). The data suggest that IKKβ stimulates a direct interaction between AhR and RORγt by inducing RORγt phosphorylation. Conversely, PLA data showed that IKKβ KO abolished the GLK-induced interaction between AhR and RORγt in T cells of Lck-GLK Tg mice (Fig. 5A). In addition, confocal imaging analysis showed that IKKβ KO specifically abolished nuclear translocation of RORγt, but not AhR, in GLK Tg T cells (Fig. 5B). Consistently, serum IL-17A levels were also decreased in Lck-GLK;IKKβ^f/f^;CD4-Cre mice compared to those in Lck-GLK Tg mice (Fig. 5C). Next, we studied whether IKKβ interacts directly with RORγt. GST and His pulldown assays using purified recombinant GST-tagged IKKβ and His-tagged RORγt proteins showed a direct interaction between IKKβ and RORγt (Fig. 5D).

To probe whether IKKβ phosphorylates RORγt, we individually immunoprecipitated Flag-tagged RORγt, IKKβ, and IKKβ kinase-dead (K44M) mutant from HEK293T transfectants and then subjected them to in vitro kinase assays. The data showed that IKKβ, but not IKKβ-K44M mutant, induced RORγt phosphorylation in vitro (Fig. 5E). To identify the IKKβ-targeted RORγt phosphorylation site, we isolated in vitro phosphorylated Flag-tagged RORγt, followed by mass spectrometry (MS) analyses. Ser^489^ was identified as the RORγt phosphorylation site by IKKβ (Fig. 5F). To demonstrate phosphorylation of the RORγt Ser^489^ site, we generated an anti–phospho-RORγt (Ser^489^) antibody, which specifically recognized RORγt wild type but not S489A mutant when cotransfected with IKKβ (Fig. 5G). Immunoblotting using this phospho-antibody showed that RORγt Ser^489^ phosphorylation was induced in T cells of Lck-GLK Tg mice, and the phosphorylation was abolished by IKKβ KO (Fig. 5H). Consistently, RORγt-S489A mutant failed to interact with AhR under IKKβ overexpression (Fig. 5I). Together, in GLK-overexpressing T cells, IKKβ phosphorylates RORγt at Ser^489^ and induces its interaction with AhR, which then transports RORγt into the nucleus and cooperates with RORγt to stimulate IL-17A transcription.

### Phosphorylated RORγt interacts with AhR in TCR-induced or murine autoimmune T cells

**Fig. 6:**
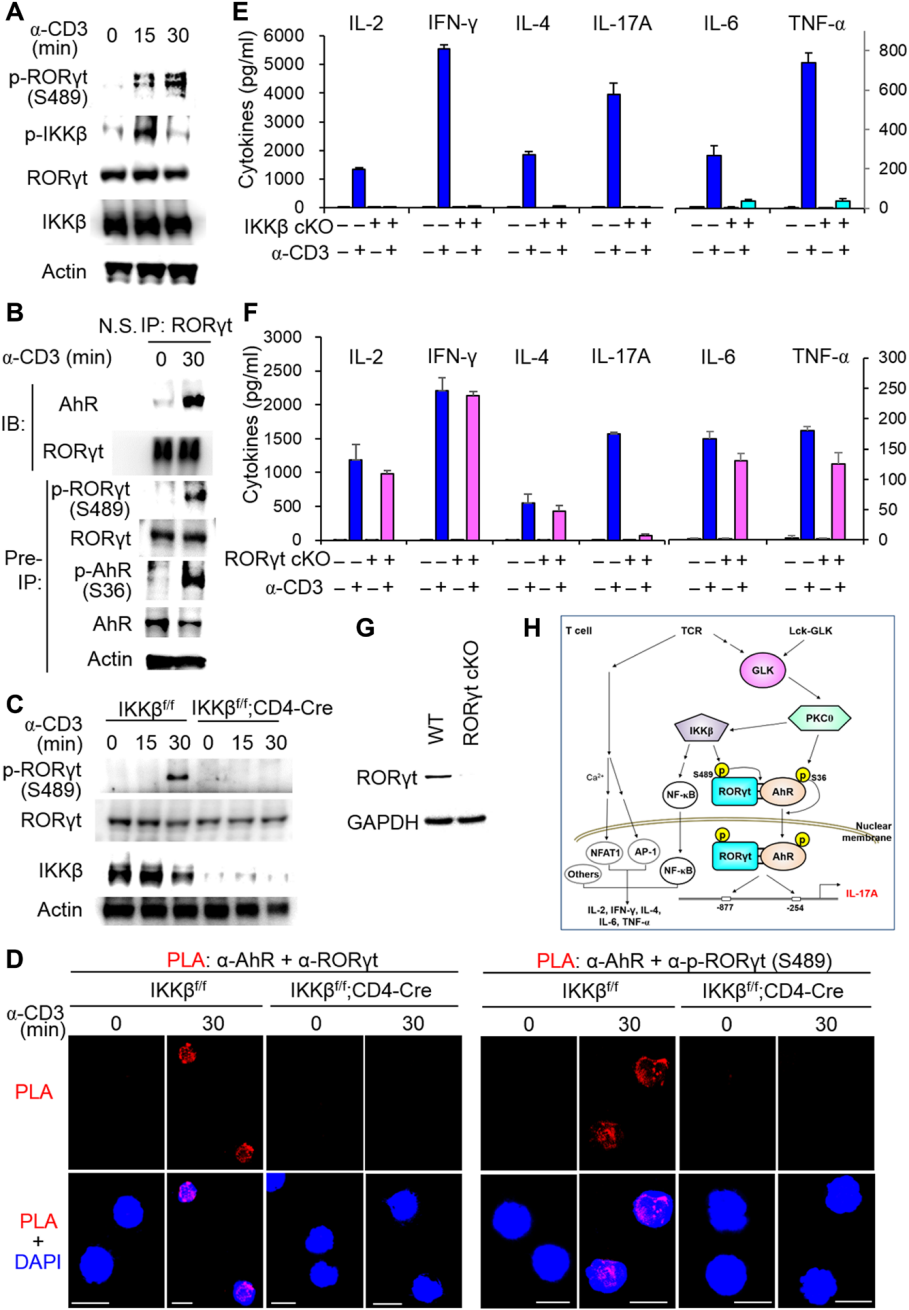
TCR signaling induces RORγt phosphorylation and subsequent AhR-RORγt interaction. (**A**) Immunoblotting analysis of p-RORγt (Ser^489^), RORγt, p-IKKβ (Ser^180/181^), and IKKβ in primary splenic T cells. T cells were stimulated with anti-CD3 antibodies plus streptavidin (3 μg each per milliliter). (**B**) Coimmunoprecipitation of endogenous AhR with RORγt from lysates of murine primary splenic T cells stimulated with anti-CD3 antibodies plus streptavidin (3 μg each per milliliter). (**C**) Immunoblotting analysis of p-RORγt (Ser^489^), RORγt, and IKKβ in primary splenic T cells of IKKβ^f/f^ or CD4-Cre;IKKβ^f/f^ mice. T cells were stimulated with anti-CD3 antibodies plus streptavidin (3 μg each per milliliter). (**D**) Confocal microscopy analysis of PLAs for the interaction between endogenous AhR and RORγt (left) or between AhR and Ser^489^-phosphorylated RORγt (right) in primary T cells of IKKβ^f/f^ or IKKβ^f/f^;CD4-Cre mice. T cells were stimulated as in (C). Each red dot represents a direct interaction. T cell nucleus was stained with DAPI (blue). Original magnification, ×630; scale bars, 10 μm. (**E** and **F**) ELISA of various cytokines in supernatants of primary splenic T cells from IKKβ^f/f^ or IKKβ^f/f^;CD4-Cre mice (E), as well as RORγt^f/f^ or RORγt^f/f^;CD4-Cre mice (F). T cells were stimulated with plate-bound anti-CD3 antibodies (2 μg each per milliliter) for 3 days. Means ± SD are shown. *n* = 3 per group. (**G**) Immunoblotting of RORγt and GAPDH proteins from primary splenic T cells of RORγt^f/f^ or RORγt^f/f^;CD4-Cre mice. Data shown (A to G) are representative of three independent experiments. (**H**) Schematic model of IL-17A transcription induced by the AhR-RORγt complex in GLK-overexpressing or TCR-stimulated T cells. GLK overexpression in T cells of T cell–specific GLK Tg (Lck-GLK Tg) mice induces AhR Ser^36^ phosphorylation through PKCθ and also induces RORγt Ser^489^ phosphorylation through IKKβ. Once RORγt is phosphorylated, RORγt interacts directly with AhR. Phosphorylated AhR is responsible for transporting RORγt into cell nucleus. The AhR-RORγt complex binds to both the RORγt-binding element (−877 to −872) and the AhR-binding element (−254 to −249) of the IL-17A promoter, leading to induction of IL-17A transcription. In normal T cells, TCR stimulation also induces GLK kinase activity and downstream signaling, including IKKβ activation, RORγt Ser^489^ phosphorylation, and the AhR-RORγt interaction. Besides NF-κB, other critical transcription factors [such as nuclear factor of activated T cell 1 (NFAT1) or activator protein 1 (AP-1)] are also required for the transcriptional activation of IL-2, IFN-γ, IL-4, IL-6, and TNF-α in T cells. “Others” denotes other critical transcription factors (table S1). NF-κB is required for TCR-induced production of multiple cytokines; however, the GLK–IKKβ–NF-κB cascade alone is not sufficient for the induction of multiple cytokines. Collectively, GLK overexpression or TCR signaling induces IL-17A transcription through AhR and RORγt in T cells.

Because both GLK and IKKβ (*24*) activation and IL-17A production (*31*, *32*) are inducible by TCR signaling, we asked whether phosphorylated RORγt-mediated AhR-RORγt interaction is also induced by TCR signaling. We found that, following IKKβ activation, anti-CD3 stimulation induced RORγt Ser^489^ phosphorylation in murine T cells (Fig. 6A). Moreover, coimmunoprecipitation assays and PLA showed that TCR signaling induced the interaction between endogenous RORγt and AhR (Fig. 6B and fig. S8A). TCR signaling also stimulated the interaction between AhR and Ser^489^-phosphorylated RORγt (fig. S8B). Conversely, IKKβ cKO abolished the TCR-induced RORγt Ser^489^ phosphorylation (Fig. 6C) and the AhR-RORγt interaction (Fig. 6D) in T cells. These data suggest that IKKβ-mediated RORγt phosphorylation and subsequent AhR-RORγt interaction are induced during T cell activation. To investigate whether IKKβ-mediated RORγt phosphorylation regulates IL-17A production after TCR stimulation, we determined secreted cytokines from anti-CD3–stimulated T cells by ELISA. Consistent with previous reports (*31*, *32*), TCR signaling induced IL-17A production in T cells (Fig. 6, E and F). IKKβ cKO abolished TCR-induced IL-17A production in T cells (Fig. 6E), supporting the notion that TCR-activated IKKβ induces IL-17A production in normal T cells. As expected, TCR-induced levels of several IKKβ/NF-κB–mediated cytokines (IL-2, IFN-γ, IL-4, IL-6, and TNF-α) (table S1) were also reduced in T cells of IKKβ cKO mice (Fig. 6E). To further verify the IKKβ–RORγt–IL-17A pathway, we used primary splenic T cells from T cell–specific RORγt cKO mice. Unlike IKKβ cKO, RORγt cKO only abolished IL-17A production upon TCR stimulation (Fig. 6F). We verified the abolishment of RORγt expression in T cells of RORγt cKO mice by immunoblotting analysis (Fig. 6G). The data suggest that IKKβ-RORγt activation mainly induces IL-17A production during TCR signaling, while IKKβ–NF-κB activation regulates the production of multiple cytokines, including IL-2, IFN-γ, IL-4, IL-6, and TNF-α (Fig. 6H and fig. S9).

## DISCUSSION

IL-17A is a major proinflammatory cytokine enhanced in the sera of patients with autoimmune diseases. Identification of upstream kinases and transcriptional mechanisms for IL-17A production should facilitate development of novel therapeutic approaches for IL-17A–mediated diseases. Here, we report that GLK, PKCθ, and IKKβ are key kinases controlling IL-17A transcription through a bifurcate signaling cascade in pathogenic T_H_17 cells.

A key finding of this report indicates that RORγt is a novel target of IKKβ; IKKβ directly phosphorylates RORγt at Ser^489^, leading to IL-17A induction. To our knowledge, this IKKβ-phosphorylated Ser^489^ is the first identified pathogenic phosphorylation site of RORγt. IL-17A induction is concomitant with increased RORγt mRNA levels under T_H_17 polarization conditions in vitro (*28*, *33*, *34*); however, RORγt mRNA levels are unchanged during TCR-induced IL-17A induction (*32*). Because of the strong induction of RORγt expression during in vitro T_H_17 differentiation (*28*, *33*, *34*), one might think that RORγt proteins are not expressed before T_H_17 differentiation. However, RORγt mRNA levels in murine T_H_0 cells are detectable, although very low (*35*, *36*). Moreover, RORγt proteins in human T_H_0 cells (*36*) or murine naïve CD4^+^ T cells (*37*–*39*) are detectable by immunoblotting. The RORγt protein levels in naïve CD4^+^ T cells are one-fifth of those in T_H_17 cells (*38*). Furthermore, data using RORγt-GFP reporter mice and flow cytometry also show that some CD3^+^ T cells in the spleen, lymph nodes, bone marrow, lung, and skin express RORγt proteins (*40*), and less than 50% of these infiltrating RORγt^+^ T cells express IL-17A (*40*). These data indicate that RORγt proteins are expressed in naïve T cells. Collectively, either overexpression or Ser^489^ phosphorylation of RORγt can lead to IL-17A transcriptional activation. Ser^489^-phosphorylated RORγt interacts with AhR and is transported into the nucleus by AhR. Notably, both TCR-stimulated or autoimmune T cells displayed induction of RORγt Ser^489^ phosphorylation and AhR-RORγt interaction. These findings suggest that IKKβ-mediated RORγt Ser^489^ phosphorylation is a common transcriptional activation mechanism of IL-17A induction in T cell activation or autoimmune diseases. Ubiquitination of RORγt at Lys^446^ negatively regulates IL-17A, but not IL-17F, production by blocking its interaction with the coactivator SRC1 (*41*). In contrast, RORγt K63-linked ubiquitination at Lys^69^ is required for IL-17A transcription (*42*). In addition, CD5 signaling may contribute to RORγt nuclear translocation in T_H_17 differentiation in vitro (*43*). IL-6 plus IL-23 stimulation also induces RORγt nuclear translocation in murine neutrophils in vitro (*44*). Thus, it is likely that RORγt Ser^489^ phosphorylation also cross-talks with or is involved in other signaling events.

One of the exciting findings in this report is that AhR is responsible for nuclear translocation of RORγt. PKCθ directly activates AhR by phosphorylating AhR at Ser^36^ during TCR signaling and GLK signaling. Ser^36^ phosphorylation of AhR mediates the nuclear translocation of the AhR-RORγt complex, and both transcription factors then induce IL-17A transcription. Paradoxically, GLK overexpression only enhanced AhR (but not RORγt) response element–driven reporter activity. Nevertheless, the mutation of either AhR-binding site or RORγt-binding site within the IL-17A promoter blocked GLK signaling–induced IL-17A transcription. It is likely that, besides nuclear translocation of RORγt by AhR, the binding of AhR to the AhR response element or indirectly binding to other regions within the IL-17A promoter may also facilitate the binding of RORγt to the IL-17A promoter. The binding of AhR with its ligand triggers the nuclear translocation and DNA binding activity of AhR; distinct ligands stimulate different physiological functions of AhR in T cells (*45*, *46*). AhR activation by a natural ligand results in enhanced T_H_17 population and severe EAE symptoms in mice (*45*, *47*). Besides TCR signaling, it is possible that AhR Ser^36^ phosphorylation may also regulate IL-17A production in ligand-induced or other unliganded signaling pathways.

GLK is required for TCR signaling and production of several cytokines such as IFN-γ, IL-4, and IL-2 (*24*); however, Lck-GLK Tg mice showed selective overproduction of IL-17A, but not other NF-κB–mediated cytokines. This dichotomy may be attributed to the fact that, besides NF-κB, other transcription factors (such as NFAT1 or AP-1) are also required for the transcriptional activation of IL-2, IL-4, IFN-γ, IL-6, or TNF-α during TCR signaling (table S1) (*48*, *49*). Thus, activation of NF-κB alone without activation of other critical transcription factors in T cell is not sufficient to induce the production of IL-2, IL-4, IFN-γ, IL-6, or TNF-α (Fig. 6H). T cell–specific IKKβ Tg mice do not display any significant induction of IL-2 or IFN-γ in resting T cells, while basal levels of the T cell activation markers CD25 and CD69 are enhanced (*50*). Consistently, activated IKKβ in GLK Tg T cells is not sufficient to induce NF-κB–mediated cytokines. In addition, STAT3 activation is required for the transcription of IL-17F, IL-21, IL-22, and IL-23R in T_H_17 cells (*39*, *51*–*53*); conversely, IL-6 or IL-21 induces STAT3 activation in T_H_17 cells (*39*, *51*, *53*). It is likely that the reason for the lack of IL-17F/IL-21/IL-22 induction and IL-23R overexpression in GLK Tg T cells may be the lack of STAT3 activation. Besides IL-17A, IL-17F and IL-22 are also regulated by RORγt (*53*). However, the Lys^466^-ubiquitinated RORγt regulates the production of IL-17A, but not IL-17F (*41*). Our results in this study showed the binding of the AhR-RORγt complex to the −877, but not the −120, region of the IL-17A promoter. These findings suggest that the AhR-RORγt complex does not bind to the RORγt-binding elements that regulate the transcription of IL-17F and IL-22. Collectively, the GLK-IKKβ-RORγt axis is not sufficient to induce multiple cytokines because of the lack of activation of other critical transcription factors or the lack of AhR-RORγt complex–binding elements. Last, it is also plausible that GLK overexpression may suppress the production of multiple cytokines through an unknown inhibitory mechanism.

Our study reveals a critical pathogenic mechanism of GLK-induced IL-17A transcription in autoimmune diseases. In summary, GLK signaling induces Ser^36^ phosphorylation and nuclear translocation of AhR through PKCθ. AhR interacts with RORγt and transports RORγt into the nucleus of T cells. The GLK-PKCθ-AhR axis is responsible for the nuclear translocation of RORγt, but not for the interaction of RORγt with AhR. On the other hand, the interaction between RORγt and AhR is controlled by IKKβ, which phosphorylates RORγt at Ser^489^. Similarly, TCR signaling also induces phosphorylation of AhR and RORγt, as well as the interaction and nuclear translocation of the AhR-RORγt complex. Thus, TCR or GLK signaling induces IL-17A transcription through the novel RORγt Ser^489^ phosphorylation and subsequent AhR-RORγt interaction/nuclear translocation. Collectively, the GLK-PKCθ/IKKβ-AhR/RORγt pathway plays a critical role in the pathogenesis of IL-17A–mediated autoimmune diseases (Fig. 6H and fig. S9). This pathway may also be involved in other IL-17A–mediated inflammatory diseases that are induced by other IL-17A^+^ cell types (for example, group 3 innate lymphoid cells). Thus, inhibitors of GLK or the AhR-RORγt complex could be used as IL-17A–blocking agents for the treatment of IL-17A–mediated diseases. Furthermore, since GLK overexpression is also correlated with cancer recurrence (*54*, *55*), studying the potential involvement of GLK-IL-17A signaling in cancer recurrence/metastasis may help future development of cancer therapy.

## METHODS

### Mice

All animal experiments were performed in the Association for Assessment and Accreditation of Laboratory Animal Care International (AAALAC)–accredited animal housing facilities at the National Health Research Institutes (NHRI). All mice were used according to the protocols and guidelines approved by the Institutional Animal Care and Use Committee of NHRI. Floxed AhR mice (JAX 006203), IL-17A–deficient mice (JAX 016879), floxed RORγt mice (JAX 008771), and floxed IKKβ mice (EMMA 001921) were purchased from the Jackson Laboratory (JAX) or the European Mouse Mutant Archive (EMMA). The three aforementioned mouse lines were backcrossed for 10 generations onto the C57BL/6 background. The data presented in this study were performed on sex-matched, 4- to 26-week-old littermates. For T cell development analyses, 5-week-old, sex-matched mice were used. All mice used in this study were maintained in temperature-controlled and pathogen-free cages.

### Generation of Lck-GLK Tg mice and PKCθ KO mice

A full-length human GLK coding sequence was placed downstream of the proximal Lck promoter (fig. S1A). Lck-GLK Tg mice in C57BL/6 background were generated using pronuclear microinjection by NHRI Transgenic Mouse Core. Two independent Lck-GLK Tg mouse lines were used. Lck-GLK Tg mouse line #1 was used in all the Lck-GLK Tg experiments, except for the studies in figs. S1B and S2E, in which Lck-GLK Tg mouse line #2 was used. PKCθ KO mice were generated by TALEN-mediated gene targeting (fig. S7, A to C); the nucleotides 5′-GGTGGAACACTAAAAATAATATGTCTTAGAGCCCCATACATACAGTGTTTGTCTTTTGTCATTTTTCTAGGGAACAACCATGTCACCGTTTC-3′ of the PKCθ intron 1 and exon 2 were deleted in the mutated allele. For TALEN-mediated gene targeting in mice, embryo microinjection of TALEN mRNA was performed by NHRI Transgenic Mouse Core.

### Cells

Human Jurkat T leukemia cells [American Type Culture Collection (ATCC), TIB-152] were cultured in RPMI 1640 (Invitrogen) containing 10% fetal calf serum (FCS; Invitrogen) plus penicillin (10 U/ml) and streptomycin (10 mg/ml) (Invitrogen). HEK293T cells (ATCC, CRL-11268) were cultured in Dulbecco’s modified Eagle’s medium (Invitrogen) containing 10% FCS plus penicillin (10 U/ml) and streptomycin (10 mg/ml). All cell lines used were tested and confirmed to be negative for mycoplasma. Primary murine T cells were negatively selected from the spleen, lymph nodes, or peripheral bloods of mice using magnetically coupled antibodies against CD11b, B220, CD49b, CD235, and TER-119 [magnetic cell separation kit (Miltenyi Biotec)]. To induce IL-17A production of 3-day–cultured T cells (Fig. 1E), the primary T cells were stimulated with biotin-conjugated anti-CD3 antibodies (3 μg/ml) plus streptavidin (3 μg/ml) for 3 hours at 37°C.

### Reagents and antibodies

GLK antibody (α–GLK-N) was generated by immunization of rabbits with peptides (murine GLK epitope: ^4^GFDLSRRNPQEDFELI^19^; identical to human GLK protein sequences 4 to 19) and was used for Figs. 2E, 3 (D and E), and 4F. Anti-GLK monoclonal antibody (clone C3) was generated by immunization of mice with peptides (murine GLK epitope: ^514^EQRGTNLSRKEKKDVPKPI^533^) and was used for Fig. 3 (F and I) and fig. S5D. The antibody for phosphorylated RORγt Ser^489^ was generated by immunization of a rabbit with phosphopeptides (murine RORγt epitope: ^483^FSTDVE[pS]PEGLSK^495^). Anti-Myc (clone 9E10), anti-Flag (clone M2), and anti-HA (clone 12CA5) antibodies were purchased from Sigma-Aldrich. Anti–p-AhR (Ser^36^; #A0765) antibody was purchased from Assay Biotechnology. Anti-AhR (clone RPT9), anti–p-IKKβ (Ser^180/181^; #ab55341), and anti-GAPDH (clone mAbcam 9484, catalog #ab9482) antibodies were purchased from Abcam. Anti-PKCθ (#3551-1) and anti-actin (clone E184) antibodies were purchased from Epitomics. Anti–p-PKCθ (Thr^538^; #ab63365), anti–p-STAT3 (Tyr^705^; clone D3A7), anti-IKKα (#2682), anti-IKKβ (#2678), anti–p-SGK1 (Thr^256^; #2939), anti-SGK1 (clone D27C11), anti-histone 3 (#9715S), anti-IRF4 (#4964S), and anti-BATF (#8638S) antibodies were purchased from Cell Signaling Technology. Anti-STAT3 (#06-596) and anti-RORγt (clone 6F3.1) antibodies were purchased from Millipore. Anti-KLF4 (#AF3158) and anti–IL-23 receptor (#bs-1460) antibodies were purchased from R&D Systems and Bioss antibodies, respectively.

### Plasmids and recombinant proteins

The expression plasmids for GLK and GLK kinase-dead mutant (GLK-K45E) were reported previously (*22*). CFP-tagged PKCθ, yellow fluorescent protein (YFP)–tagged AhR, Myc-tagged PKCθ, HA-tagged AhR, and Flag-tagged PKCθ plasmids were constructed by subcloning individual complementary DNAs into pCMV6-AC-CFP, pCMV6-AC-YFP, pCMV6-AC-Myc, pCMV6-AC-HA, or pCMV6-AC-Flag vector (OriGene Technologies). HA-tagged AhR-S36A mutant and Myc-tagged PKCθ-K409W (kinase-dead) mutant plasmids were generated by site-directed mutagenesis. GST-tagged PKCθ and GST-tagged PKCθ-K409W plasmids were also individually constructed by subcloning into pGEX-4T vector. The human GLK shRNA (5′-GTGCCACTTAGAATGTTTGAAA-3′) was obtained from the National RNAi Core Facility (Taiwan) and subcloned into pSUPER-GFP vector (Oligoengine). The IL-17A reporter plasmid was a gift from W. Strober (Addgene plasmid #20124) (*28*). The IL-17A promoter constructs containing a mutated binding element for AhR, RORγt (−877), RORγt (−120), or STAT3 were generated by site-directed mutagenesis using Fusion DNA polymerase (Thermo Fisher Scientific) according to the manufacturer’s protocol. The following primers were used for mutagenesis (mutated binding elements are underlined and italicized; mutated nucleotides are shown in bold font): AhR-binding site (*18*), 5′-ATGTCCATACA***T***ACATGATACTGAATCACAGC-3′; RORγt-binding site (−887) (*28*), 5′-CTCAAAGACATAAAGGCAA***CC***GT***G***ATCTCATGGAGAGGAGAG-3′; RORγt-binding site (−120), 5′-GGTTCTGTGCTG***CAA***TCATTTGAGG-3′ (*11*); and STAT3-binding site (*56*), 5′-AGACAGATGTTGCC***T***GTCATAAAGGGGTGGTT-3′.

The mutant constructs were verified by DNA sequencing. The plasmids pGL4.43-Luc2P-XRE (AhR-response XRE-Luc), pGL4.47-Luc2P-SIE (STAT3-responsive SIE-Luc), pGL4.32–Luc2P–NF-κB–RE–Hygro, and pGL4 luciferase reporter vector were purchased from Promega. The plasmid for the RORγt (−877) response element–driven reporter was constructed by cloning four copies of the RORγt (−877) response element into the pGL4.43 luciferase reporter vector. For in vitro binding assays, purified AhR proteins were isolated from HA-AhR–transfected HEK293T cells, followed by HA-peptide elution. Purified recombinant GST-PKCθ and GST-IKKβ proteins were purchased from SignalChem. Purified recombinant 6×His-RORγ proteins were purchased from MyBioSource. Recombinant proteins of GST-tagged PKCθ K409W proteins were isolated from *Escherichia coli* (BL21) and then purified by GST pulldown assays. Purified Flag-tagged RORγt protein was immunoprecipitated and then eluted with Flag peptides from lysates of HEK293T cells that were cotransfected with Flag-RORγt plus either CFP-IKKβ or vector. Purified recombinant protein of GST-tagged AhR was purchased from Abnova.

### Luciferase reporter assays

The 2-kb IL-17A promoter–driven firefly luciferase reporter plasmid and a *Renilla* luciferase control plasmid (pRL-TK) were cotransfected into Jurkat T cells. After 24 hours, 1 × 10^6^ cells were harvested, washed with phosphate-buffered saline, and resuspended in 60 μl of RPMI 1640 plus 60 μl of lysis buffer. Data represent the mean of the ratios of the firefly luciferase activity to the *Renilla* luciferase activity.

### Enzyme-linked immunosorbent assays

Serum levels of IL-1β, IL-4, IL-6, IL-12, IL-17F, IL-21, IL-22, IL-23, IFN-γ, TNF-α, and TGF-β were analyzed by individual ELISA kits purchased from eBioscience. The IL-17A levels were determined using an ELISA kit from BioLegend. The serum levels of ANAs, anti-dsDNA antibody, and RF were analyzed by ELISA kits purchased from Alpha Diagnostic International.

### ALPHA technology

ALPHA technology/protein-protein interaction assays were performed according to the manufacturer’s protocol from PerkinElmer Life Sciences, as described in a previous publication (*57*). When the protein-donor pair was within 200 nm, a luminescent signal was detected by an EnVision 2104 Multilabel Plate Reader (PerkinElmer Life Sciences).

### FRET assays

FRET signal in live cells was detected by an EnVision 2104 Multilabel Plate Reader (PerkinElmer Life Sciences). The reaction was excited by light passing through a 430-nm filter (with 8 nm bandwidth), and the intensity of emitted fluorescence passing through a 530-nm filter (with 8 nm bandwidth) was recorded. If the protein-protein pair was in close proximity (1 to 10 nm), a 530-nm signal would be detected. The FRET efficiency was calculated by a formula, efficiency = (1 − FDA/FD) × 100% (where FDA is the relative fluorescence intensities of the donor in the presence of the acceptor and FD is the relative fluorescence intensities of the donor in the absence of the acceptor).

### In situ PLA

PLAs were performed using the Duolink In Situ Red Starter kit (Sigma-Aldrich) according to the manufacturer’s instructions. Briefly, cells were incubated with rabbit or mouse primary antibodies for each molecule pair (AhR plus PKCθ, AhR plus RORγt, or Flag plus Myc), followed by species-specific secondary antibodies conjugated with oligonucleotides (PLA probes). After ligation and amplification reactions, the PLA signals from each pair of PLA probes in close proximity (<40 nm) were visualized as individual red dots by a fluorescence microscope (Leica DM2500) or a confocal microscope (Leica TCS SP5 II). Each red dot represents a direct interaction.

### ChIP assays

Peripheral blood T cells of mice were cross-linked with 1% formaldehyde for 10 min at room temperature. The lysates were sonicated (3 × 9 s) on ice to generate 200– to 1000–base pair DNA fragments. The cell extracts were immunoprecipitated with 5 μg of anti-RORγt, anti-AhR, or anti-STAT3 antibodies plus protein G Dynabeads (Invitrogen) for 4 hours at 4°C on a rotating wheel. After washing three times, immunocomplexes were incubated at 94°C for 15 min for reverse cross-linking and then treated with protease K. The DNA fragments were purified using a PCR purification kit (GE Healthcare) and subjected to PCR for 35 cycles. The primers for the IL-17A promoter containing the RORγt-binding sites (−877 to −872) (*28*) and (−120 to −115) (*11*), AhR-binding site (−254 to −249) (*18*), STAT3-binding site (−150 to −145) (*56*), IRF4-binding site (−429 to −421) (*14*), KLF4-binding site (−1097 to −1083) (*16*), or BATF (−243 to −176) (*15*) were as follows: RORγt (−877 to −872), 5′-CTGAAGAGCTGGGACCTAATG-3′ (forward) and 5′-GACTACTAACAGGAGGAGATG-3′ (reverse); RORγt (−120 to −115), 5′-GGTTCTGTGCTGACCTCATTTGAG-3′ (forward) and 5′-CACAGATGAAGCTCTCCCTGG-3′ (reverse); AhR, 5′-GAGACTCACAAACCATTACTATG-3′ (forward) and 5′-CACAGATGAAGCTCTCCCTGG-3′ (reverse); STAT3, 5′-GAGACTCACAAACCATTACTATG-3′ (forward) and 5′-CACAGATGAAGCTCTCCCTGG-3′ (reverse); IRF4, 5′-GGGCAAGGGATGCTCTCTAG-3′ (forward) and 5′-CTGAAGCTGCTGCAGAGCTG-3′ (reverse); KLF4, 5′-GGGTATTATCCCAAGGGTATCC-3′ (forward) and 5′-ATGCAGCATGAGGTGGACCGAT-3′ (reverse); BATF, 5′-GAACTTCTGCCCTTCCCATCT-3′ (forward) and 5′-CAGCACAGAACCACCCCTTT-3′ (reverse).

### Immunoprecipitation, GST/His pulldown, and immunoblotting analyses

Immunoprecipitation was performed by preincubation of 0.5 to 1 mg of protein lysates with 1 μg of antibody for 1 hour at 4°C, followed by the addition of 20 μl of protein A/G Sepharose beads for 3 hours. For GST and His pulldown assays, GST- and His-tagged proteins plus their interacting proteins were incubated for 3 hours with glutathione Sepharose beads (GE Healthcare) and Ni Sepharose beads (GE Healthcare), respectively. The immunocomplexes or GST/His pulldown complexes were washed with lysis buffer (1.5 mM MgCl_2_, 0.2% NP-40, 125 mM NaCl, 5% glycerol, 25 mM NaF, 50 mM tris-HCl, and 1 mM Na_3_VO_4_) three times at 4°C, followed by boiling in 5× loading buffer at 95°C for 3 min. The immunoblotting analyses were performed as described previously (*57*).

### Cell transfections, T cell stimulation, in vitro kinase assays for PKCθ or IKKβ, and flow cytometric analyses

These experiments were performed as described previously (*24*).

### Immunofluorescence and confocal imaging

Cells were fixed in cold methanol for 2 min. After permeation with 0.25% Triton X-100 for 1 hour, the cells were blocked with 5% bovine serum albumin for 2 hours. The cells were incubated with primary antibodies (1:200 dilution) for 24 hours and then with secondary antibodies (1:500 dilution) for 2 hours. The secondary antibodies donkey anti-rabbit immunoglobulin G (IgG)–Alexa Fluor 568 and goat anti-mouse IgG–Alexa Fluor 488 were purchased from Abcam and Life Technologies, respectively. The cover slides were mounted in Fluoroshield with DAPI (GeneTex) and analyzed using a Leica TCS SP5 confocal microscope.

### In vitro T cell differentiation assays

CD4^+^ splenic T cells were purified from mice. Cells (5 × 10^5^) were cultured in 1 ml of RPMI 1640 in 48-well plates coated with anti-CD3 (2 μg/ml) and anti-CD28 (3 μg/ml) antibodies. For T_H_17 differentiation, splenic CD4^+^ cells were cultured in the medium containing IL-23 recombinant proteins (50 ng/ml; R&D Systems), IL-6 recombinant proteins (20 ng/ml; R&D Systems), TGF-β recombinant proteins (5 ng/ml; R&D Systems), and anti–IL-4 (5 μg/ml; BioLegend) and anti–IFN-γ (5 μg/ml; BioLegend) antibodies. For T_H_1 differentiation, CD4^+^ splenic cells were cultured in the medium containing IL-12 recombinant proteins (5 ng/ml; R&D Systems) and anti–IL-4 antibodies (1 μg/ml; BioLegend).

### Liquid chromatography–MS

After in vitro kinase assays, protein bands of Flag-tagged RORγt were collected from InstantBlue (GeneMark)–stained SDS–polyacrylamide gel electrophoresis gels. Proteins were digested with trypsin and subjected to liquid chromatography–MS/MS analyses by an LTQ Orbitrap Elite hybrid mass spectrometer as described previously (*57*).

### Statistical analyses

All experiments were repeated at least three times. Data are means ± SEM. The statistical significance between two unpaired groups was analyzed using two-tailed Student’s *t* test. *P* values of less than 0.05 were considered statistically significant.

## Supplementary Material

http://advances.sciencemag.org/cgi/content/full/4/9/eaat5401/DC1

## SUPPLEMENTARY MATERIALS

Supplementary material for this article is available at http://advances.sciencemag.org/cgi/content/full/4/9/eaat5401/DC1 Fig. S1. Normal T cell and B cell development in Lck-GLK Tg mice. Fig. S2. Inflammatory phenotypes and enhanced T_H_17 differentiation in Lck-GLK Tg mice. Fig. S3. Autoimmune responses in Lck-GLK Tg mice are abolished by IL-17A deficiency. Fig. S4. GLK transgene does not regulate IL-23 receptor expression, STAT3 phosphorylation, and RORγt-binding element at the −120 region of the IL-17A promoter. Fig. S5. PKCθ controls Ser^36^ phosphorylation–mediated AhR nuclear translocation and AhR-mediated autoimmune responses. Fig. S6. PKCθ directly interacts with AhR in the cytoplasm of Lck-GLK T cells. Fig. S7. Autoimmune responses in Lck-GLK Tg mice are reduced by PKCθ KO. Fig. S8. TCR signaling induces in vivo interaction between AhR and RORγt. Fig. S9. Schematic model of AhR/RORγt-mediated IL-17A transcription in T cells of Lck-GLK Tg mice with different gene-KO backgrounds. Table S1. Transcription factors of NF-κB–mediated cytokines. References (*58*, *59*)
