# Supplementary material for: GLK-IKKβ signaling induces dimerization and translocation of the AhR-RORγt complex in IL-17A induction and autoimmune disease
Source: Sci Adv. 2018 Sep 12;4(9):eaat5401. doi: 10.1126/sciadv.aat5401 (PMC6135549; doi:10.1126/sciadv.aat5401)
Supplement: http://advances.sciencemag.org/cgi/content/full/4/9/eaat5401/DC1 [file supp_4_9_eaat5401__index.html]

Science Advances | Science Advances

## Supplementary Materials

**This PDF file includes:**

- Fig. S1. Normal T cell and B cell development in Lck-GLK Tg mice.
- Fig. S2. Inflammatory phenotypes and enhanced TH17 differentiation in Lck-GLK Tg mice.
- Fig. S3. Autoimmune responses in Lck-GLK Tg mice are abolished by IL-17A deficiency.
- Fig. S4. GLK transgene does not regulate IL-23 receptor expression, STAT3 phosphorylation, and RORγt-binding element at the −120 region of the IL-17A promoter.
- Fig. S5. PKCθ controls Ser36 phosphorylation–mediated AhR nuclear translocation and AhR-mediated autoimmune responses.
- Fig. S6. PKCθ directly interacts with AhR in the cytoplasm of Lck-GLK T cells.
- Fig. S7. Autoimmune responses in Lck-GLK Tg mice are reduced by PKCθ KO.
- Fig. S8. TCR signaling induces in vivo interaction between AhR and RORγt.
- Fig. S9. Schematic model of AhR/RORγt-mediated IL-17A transcription in T cells of Lck-GLK Tg mice with different gene-KO backgrounds.
- Table S1. Transcription factors of NF-κB–mediated cytokines.
- References (*58*, *59*)

Download PDF

**Files in this Data Supplement:**

- Adobe PDF - aat5401\_SM.pdf
