# Supplementary material for: GLK-IKKβ signaling induces dimerization and translocation of the AhR-RORγt complex in IL-17A induction and autoimmune disease
Source: Sci Adv. 2018 Sep 12;4(9):eaat5401. doi: 10.1126/sciadv.aat5401 (PMC6135549; doi:10.1126/sciadv.aat5401)
Supplement: http://advances.sciencemag.org/cgi/content/full/4/9/eaat5401/DC1 [file aat5401_SM.pdf]

Science Advances
AAAS

advances.sciencemag.org/cgi/content/full/4/9/eaat5401/DC1

Supplementary Materials for

GLK-IKKβ signaling induces dimerization and translocation of the AhR-RORγt complex in IL-17A induction and autoimmune disease

Huai-Chia Chuang, Ching-Yi Tsai, Chia-Hsin Hsueh, Tse-Hua Tan*

*Corresponding author. Email: ttan@nhri.org.tw

Published 12 September 2018, *Sci. Adv.* **4**, eaat5401 (2018)
DOI: 10.1126/sciadv.aat5401

This PDF file includes:

Fig. S1. Normal T cell and B cell development in Lck-GLK Tg mice.
Fig. S2. Inflammatory phenotypes and enhanced T_H17 differentiation in Lck-GLK Tg mice.
Fig. S3. Autoimmune responses in Lck-GLK Tg mice are abolished by IL-17A deficiency.
Fig. S4. GLK transgene does not regulate IL-23 receptor expression, STAT3 phosphorylation, and RORγt-binding element at the −120 region of the IL-17A promoter.
Fig. S5. PKCθ controls Ser^{36} phosphorylation–mediated AhR nuclear translocation and AhR-mediated autoimmune responses.
Fig. S6. PKCθ directly interacts with AhR in the cytoplasm of Lck-GLK T cells.
Fig. S7. Autoimmune responses in Lck-GLK Tg mice are reduced by PKCθ KO.
Fig. S8. TCR signaling induces in vivo interaction between AhR and RORγt.
Fig. S9. Schematic model of AhR/RORγt-mediated IL-17A transcription in T cells of Lck-GLK Tg mice with different gene-KO backgrounds.
Table S1. Transcription factors of NF-κB–mediated cytokines.
References (*58*, *59*)

Supplementary Figures:

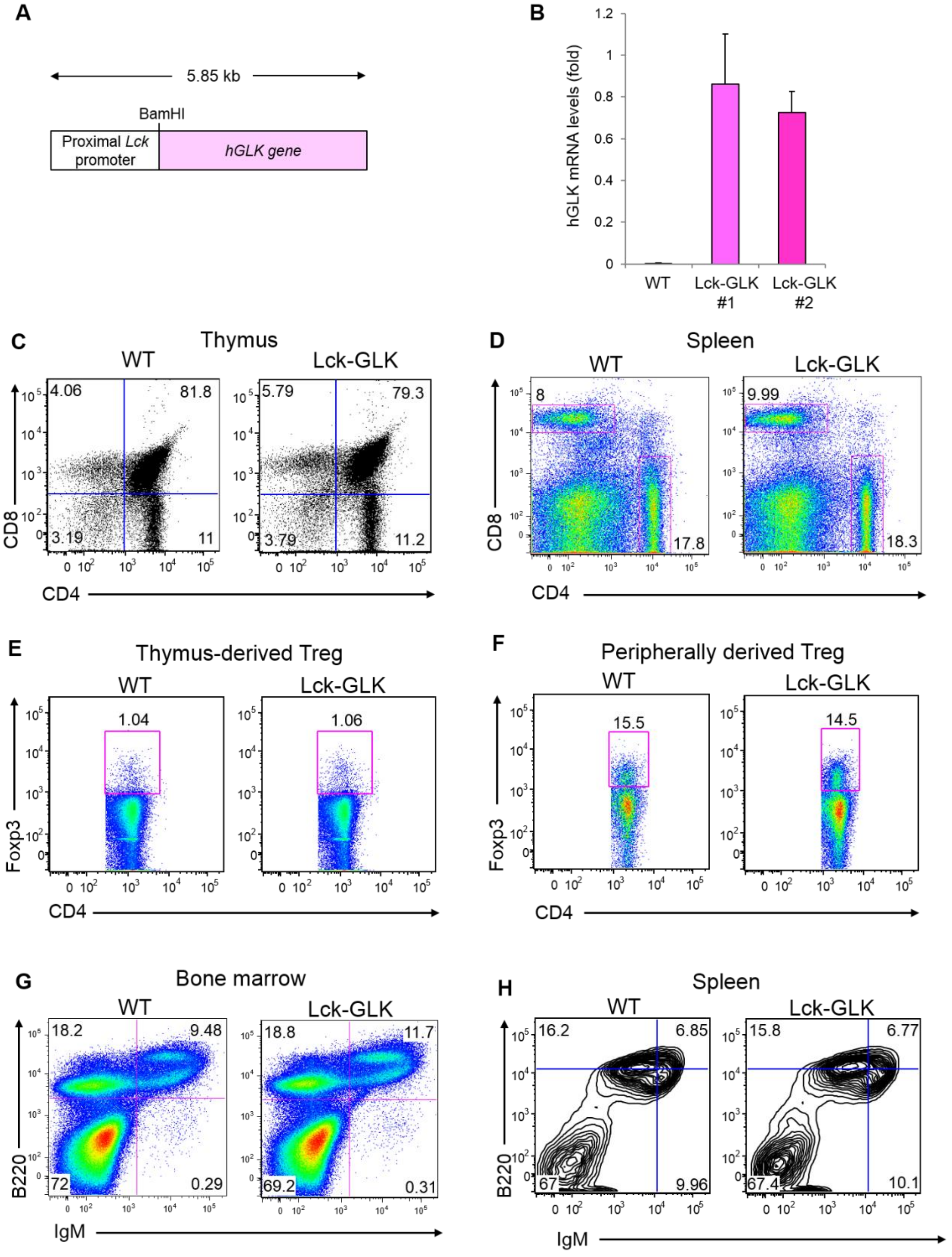

Fig. S1. Normal T cell and B cell development in Lck-GLK Tg mice. (**A**) Schematic diagram of the Lck-GLK construction. (**B**) Real-time PCR analyses of transgenic human GLK (hGLK) mRNA levels in murine splenic T cells from 5 mice per group. The expression levels of hGLK were normalized to Srp72 levels. The fold changes are presented relative to the value of wild-type mice. Means ± SEM are shown. WT, wild-type littermate controls; Lck-GLK, T-cell-specific GLK transgenic mice. #1 denotes the Lck-GLK Tg mouse line 1. #2 denotes the Lck-GLK Tg mouse line 2. (**C-F**) Flow cytometry analyses of T cells (C, D) and Treg cells (E, F) from the thymus, spleen, or peripheral blood of 5-week-old wild-type or Lck-GLK transgenic mice. (**G** and **H**) Flow cytometry analyses of $B220^+$ B cells from the bone marrow (G) or spleen (H) of wild-type or Lck-GLK transgenic mice. WT, wild-type littermate controls; Lck-GLK, T-cell-specific GLK transgenic mice. Data shown are representatives of three independent experiments.

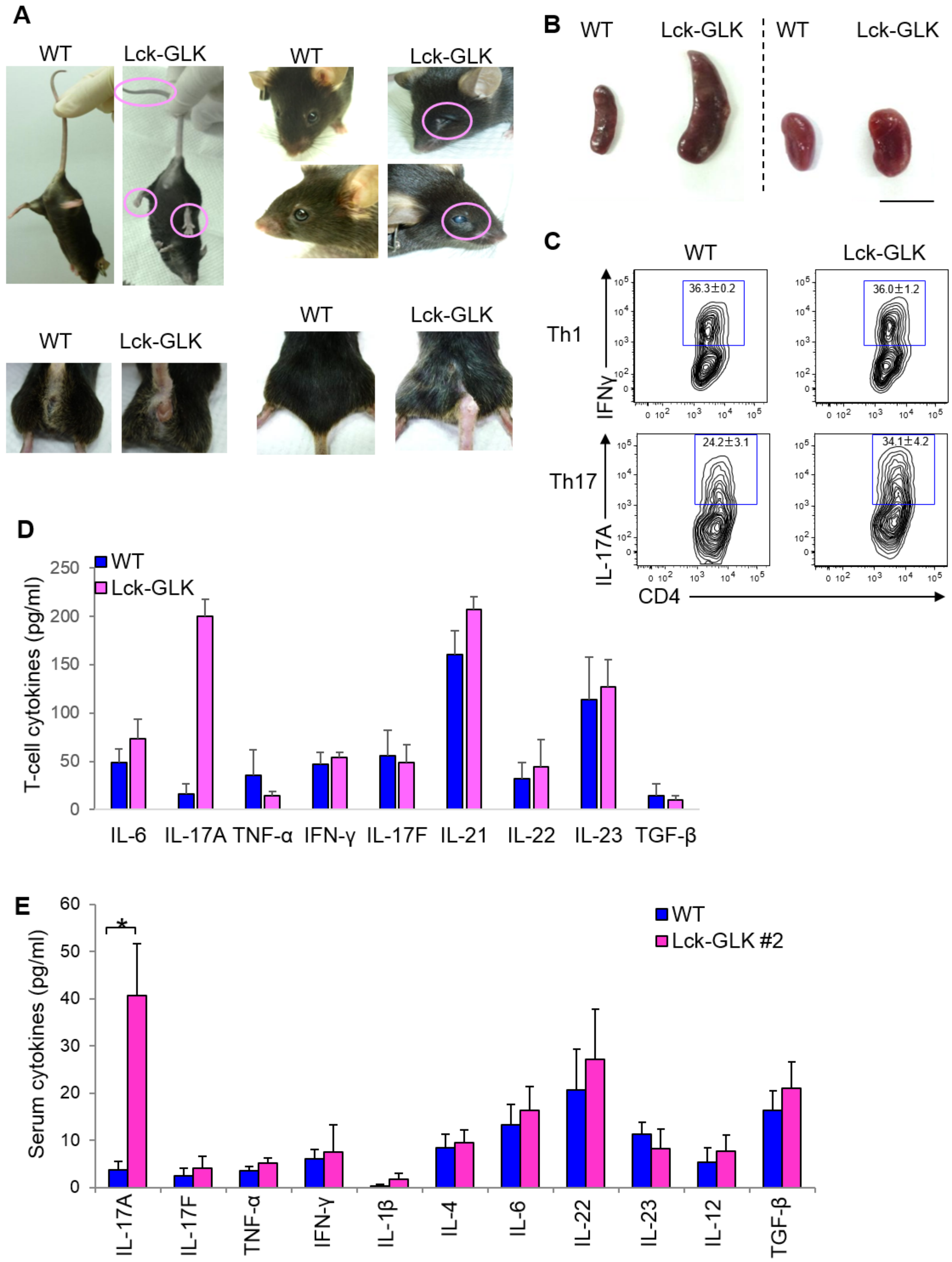

A
WT
Lck-GLK
B
WT
Lck-GLK
WT
Lck-GLK
C
WT
Lck-GLK
Th1
IFNγ
36.3±0.2
36.0±1.2
Th17
IL-17A
24.2±3.1
34.1±4.2
CD4
D
WT
Lck-GLK
T-cell cytokines (pg/ml)
IL-6
IL-17A
TNF-α
IFN-γ
IL-17F
IL-21
IL-22
IL-23
TGF-β
E
WT
Lck-GLK #2
Serum cytokines (pg/ml)
*
IL-17A
IL-17F
TNF-α
IFN-γ
IL-1β
IL-4
IL-6
IL-22
IL-23
IL-12
TGF-β

Fig. S2. Inflammatory phenotypes and enhanced T_H17 differentiation in Lck-GLK Tg mice. (**A**) Inflammatory phenotypes of 16-week-old Lck-GLK transgenic mice. The inflammatory phenotypes include paralyses of the hind-limb and tail, clouding of the eye, and symptoms of proctitis and dermatitis. (**B**) Enlargement of the spleen (left panel) and kidney (right panel) in 16-week-old Lck-GLK transgenic mice. Bar, 1 cm. WT, wild-type littermate controls; Lck-GLK, Lck-GLK transgenic mice. (**C**) Th17 but not Th1 differentiation *in vitro* is enhanced by GLK transgene. Flow cytometry of IFN-γ-producing and IL-17A-producing $CD4^+$ T cells. Data are presented as mean ± SEM. Numbers inside outlined areas indicate percent cells in each subpopulation. Data were collected with FACSCanto II (BD Biosciences) and analyzed using FlowJo software. (**D**) The cytokine levels in T-cell supernatants were determined using ELISA assays. Primary splenic T cells from wild-type or Lck-GLK Tg mice were cultured for 72 h without any stimulation. Means ± SD are shown. n = 3 per group. (**E**) The serum levels of cytokines in 4-week-old mice were determined using ELISA assays. WT, n = 10; Lck-GLK, n = 7. WT, wild-type littermate controls; Lck-GLK, T-cell-specific GLK transgenic mice. #2 denotes the Lck-GLK Tg mouse line 2. *, P value < 0.05 (two-tailed Student's t-test).

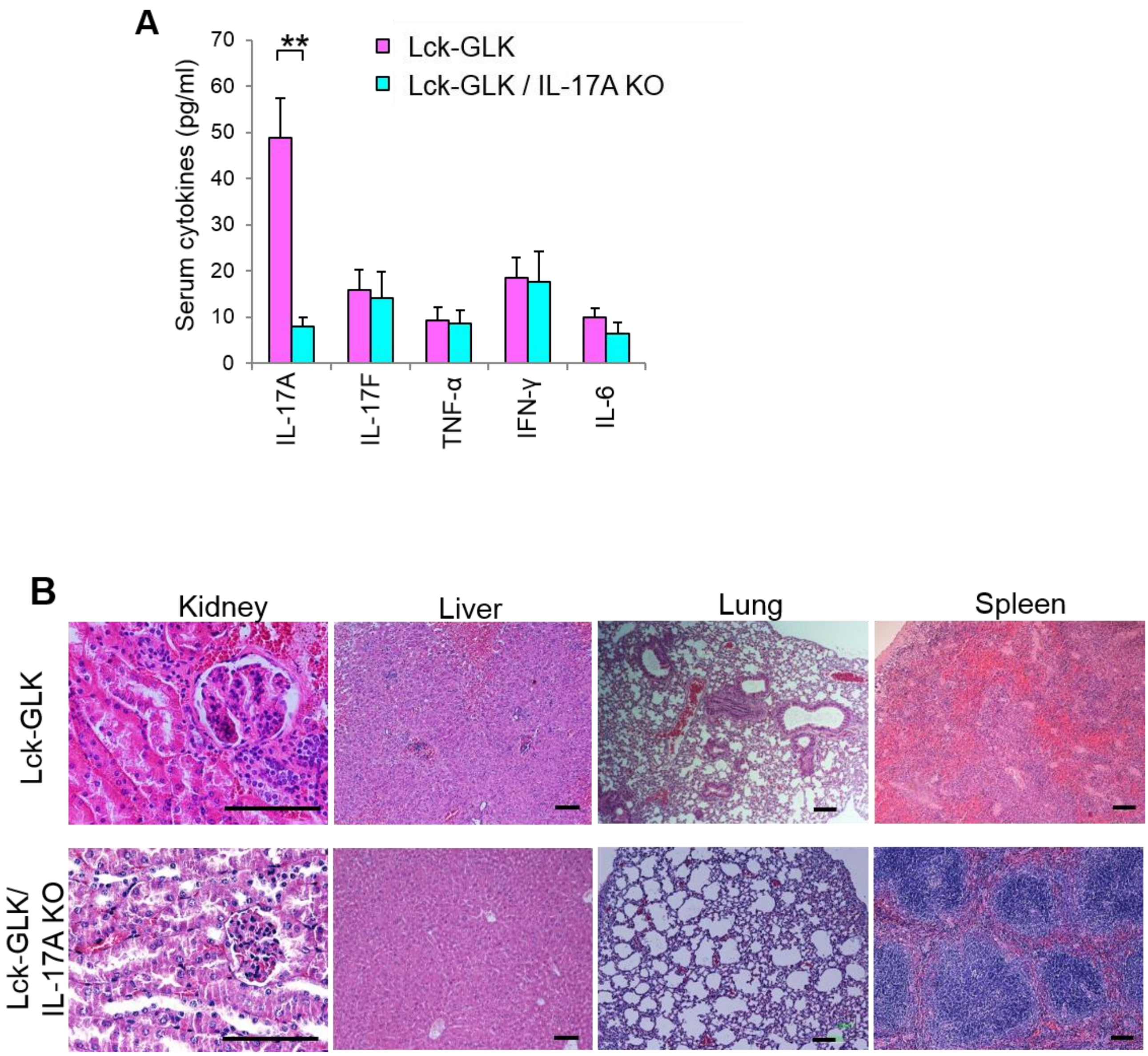

Fig. S3. Autoimmune responses in Lck-GLK Tg mice are abolished by IL-17A deficiency. (**A**) The serum levels of cytokines in 4-week-old Lck-GLK transgenic and Lck-GLK/IL-17A KO mice were determined by ELISA assays. n = 6 per group. Means ± SEM are shown. **, *P* value < 0.01 (two-tailed Student's t-test). (**B**) Hematoxylin and eosin-stained sections of indicated organs from 16-week-old mice. Bar, 100 µm. Lck-GLK, T-cell-specific GLK transgenic mice; Lck-GLK/IL-17A KO, Lck-GLK transgenic mice bred with IL-17A-deficient mice.

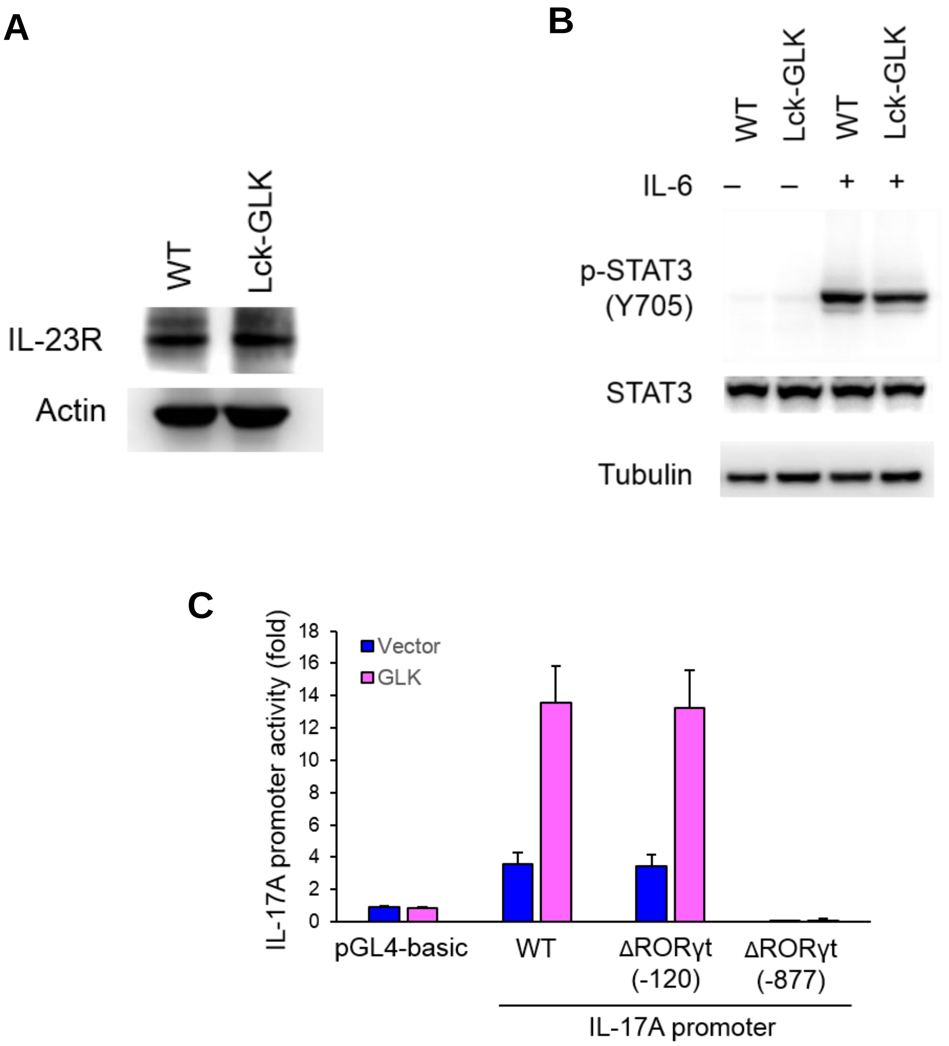

Fig. S4. GLK transgene does not regulate IL-23 receptor expression, STAT3 phosphorylation, and RORγt-binding element at the −120 region of the IL-17A promoter. (**A**) Immunoblotting analyses of IL-23 receptor (IL-23R) and Actin in splenic T cells isolated from wild-type or Lck-GLK transgenic mice. (**B**) Immunoblotting analyses of phosphorylated STAT3 (Tyr-705), STAT3, and Tubulin in unstimulated or IL-6 (1 μg/ml)-stimulated splenic T cells isolated from wild-type or Lck-GLK transgenic mice. WT, wild-type littermate controls; Lck-GLK, T-cell-specific GLK transgenic mice. Data shown (a and b) are representatives of three independent experiments. (**C**) Luciferase activity of IL-17A reporter assays in Jurkat T cells co-transfected with empty vector or GLK plasmid plus the IL-17A promoter construct containing a mutated binding element for either RORγt (-120) or RORγt (-877). Means ± SEM of three independent experiments are shown.

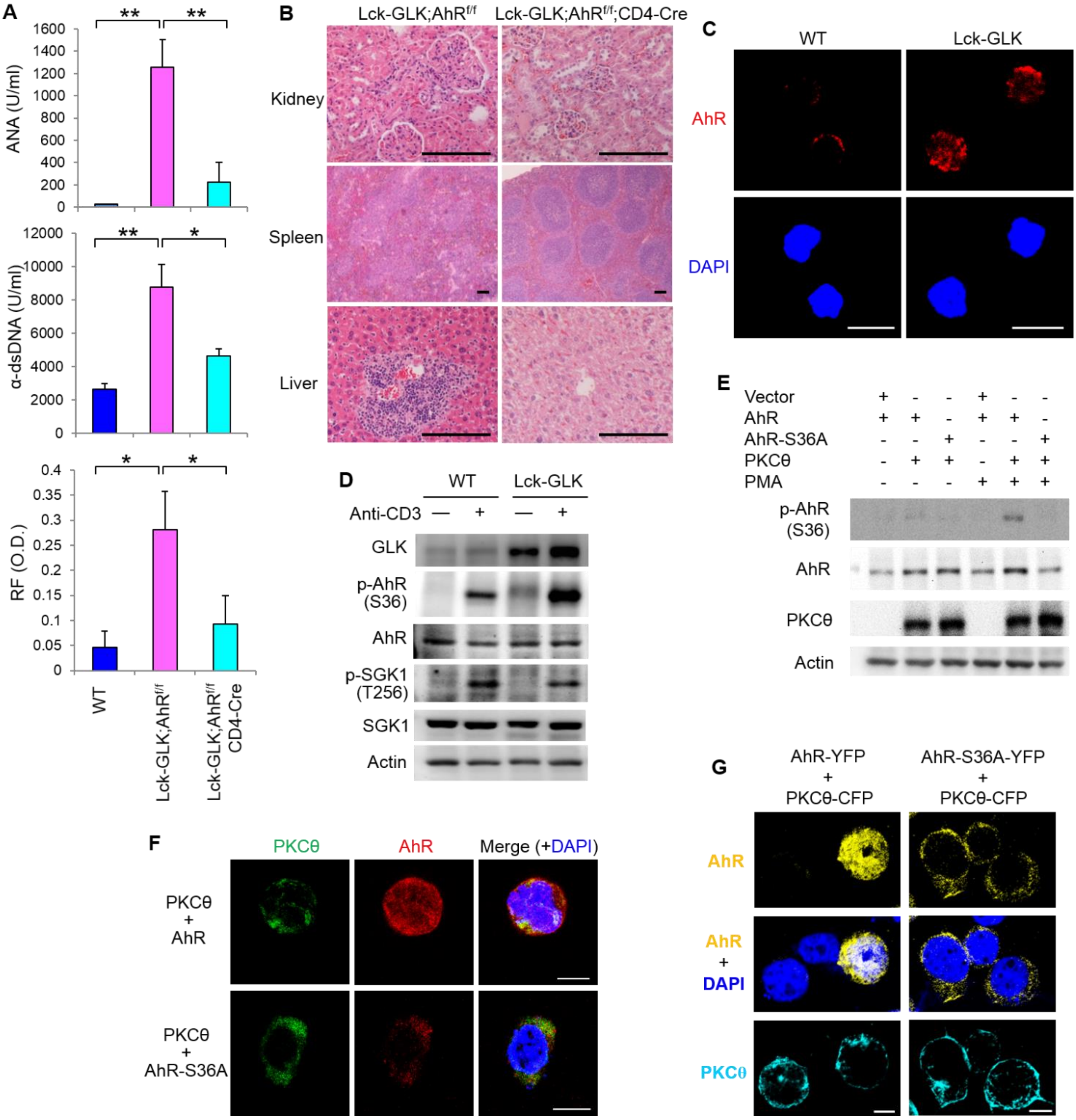

A
ANA (U/ml)
α-dsDNA (U/ml)
RF (O.D.)
WT
Lck-GLK;AhR$^{f/f}$
Lck-GLK;AhR$^{f/f}$ CD4-Cre
B
Lck-GLK;AhR$^{f/f}$
Lck-GLK;AhR$^{f/f}$;CD4-Cre
Kidney
Spleen
Liver
C
WT
Lck-GLK
AhR
DAPI
D
WT
Lck-GLK
Anti-CD3
GLK
p-AhR (S36)
AhR
p-SGK1 (T256)
SGK1
Actin
E
Vector
AhR
AhR-S36A
PKCθ
PMA
p-AhR (S36)
AhR
PKCθ
Actin
F
PKCθ
AhR
Merge (+DAPI)
PKCθ + AhR
PKCθ + AhR-S36A
G
AhR-YFP + PKCθ-CFP
AhR-S36A-YFP + PKCθ-CFP
AhR
AhR + DAPI
PKCθ

Fig. S5. PKCθ controls Ser^{36} phosphorylation–mediated AhR nuclear translocation and AhR-mediated autoimmune responses. (**A** and **B**) Attenuation of autoimmune responses in Lck-GLK transgenic mice by AhR conditional knockout. The serum levels of autoantibodies from 16-week-old mice were determined by ELISA assays (A). n = 8 per group. Means ± SEM are shown. Hematoxylin and eosin-stained sections of indicated organs from 16-week-old mice (B). (**C**) Confocal microscopy analyses of subcellular localization of AhR in murine splenic T cells without any stimulation. An anti-AhR antibody (#sc-5579, Santa Cruz) was used. Original magnification, × 630; bar, 10 µm. (**D**) Immunoblotting analyses of phosphorylated AhR (Ser-36), phosphorylated SGK1 (Thr-256), AhR, SGK1, GLK, and Actin in primary splenic T cells of wild-type and Lck-GLK transgenic mice. (**E**) Antibody specificity of the anti-phospho-AhR (Ser-36) was demonstrated by immunoblotting using HEK293T cells co-transfected with Myc-tagged PKCθ plus either HA-tagged AhR WT or AhR-S36A mutant. (**F**) Confocal microscopy analyses of subcellular localization of AhR and PKCθ in Jurkat T cells co-transfected with PKCθ plus either HA-tagged AhR WT or AhR-S36A mutant. Original magnification, × 630; bar, 10 µm. (**G**) Confocal microscopy analyses of subcellular localization of CFP-tagged PKCθ and either YFP-tagged AhR or YFP-tagged AhR-S36A mutant in co-transfected HEK293T cells stimulated with PMA. Original magnification, × 630; bar, 10 µm. Data shown are representatives of three independent experiments.

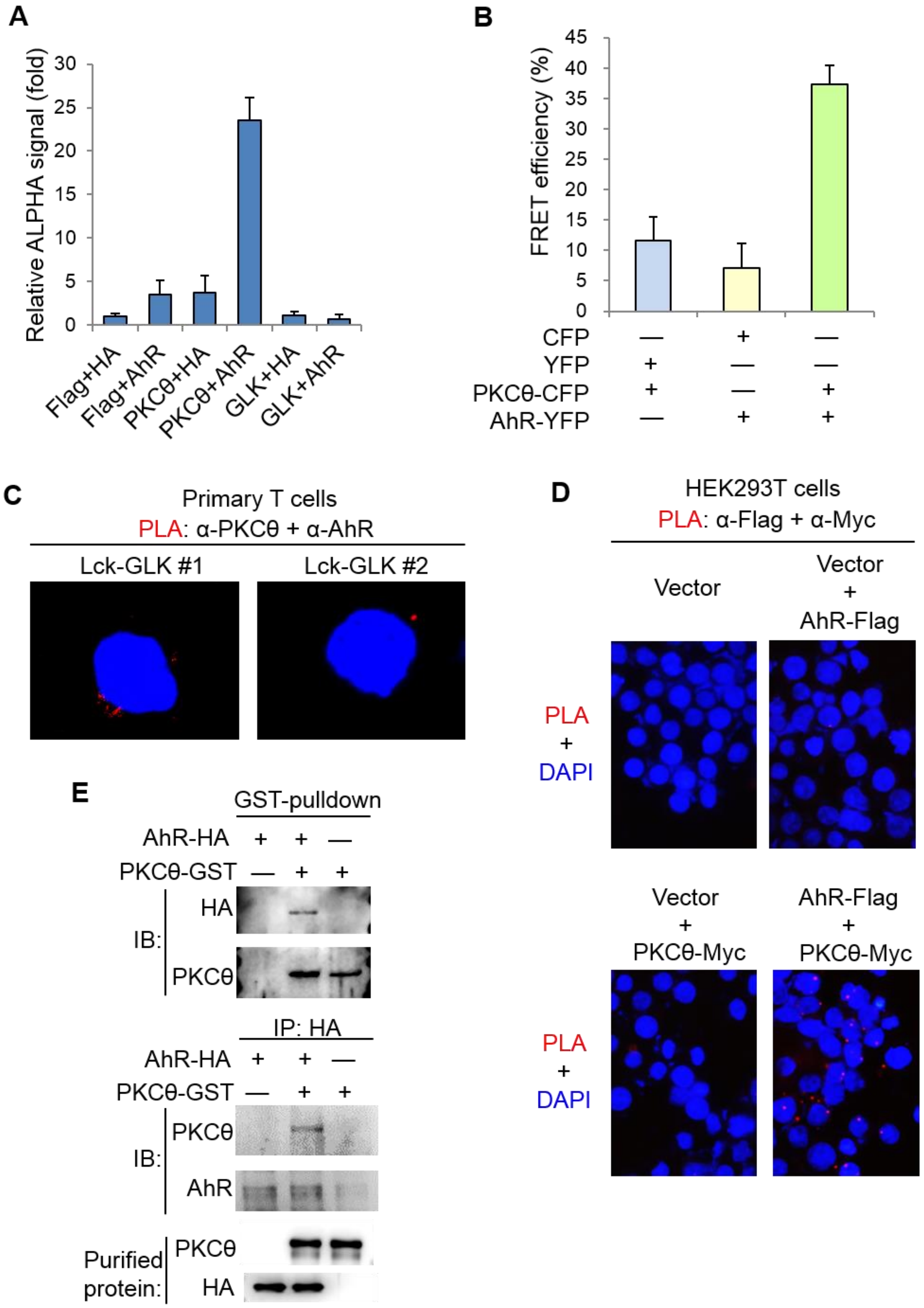
A
Relative ALPHA signal (fold)
30
25
20
15
10
5
0
Flag+HA
Flag+AhR
PKCθ+HA
PKCθ+AhR
GLK+HA
GLK+AhR
B
FRET efficiency (%)
45
40
35
30
25
20
15
10
5
0
CFP — + —
YFP + — —
PKCθ-CFP + — +
AhR-YFP — + +
C
Primary T cells
PLA: α-PKCθ + α-AhR
Lck-GLK #1
Lck-GLK #2
D
HEK293T cells
PLA: α-Flag + α-Myc
Vector
Vector + AhR-Flag
PLA + DAPI
Vector + PKCθ-Myc
AhR-Flag + PKCθ-Myc
PLA + DAPI
E
GST-pulldown
AhR-HA + + —
PKCθ-GST — + +
IB:
HA
PKCθ
IP: HA
AhR-HA + + —
PKCθ-GST — + +
IB:
PKCθ
AhR
Purified protein:
PKCθ
HA

Fig. S6. PKCθ directly interacts with AhR in the cytoplasm of Lck-GLK T cells. (**A**) Signals of the interaction (< 200 nm) between HA-AhR and Flag-PKCθ in lysates of HEK293T cells determined by amplified luminescent proximity homogeneous assays (ALPHA). Means ± SEM are shown. (**B**) Fluorescence resonance energy transfer (FRET) analysis of the direct interaction (1-10 nm) between CFP-tagged PKCθ and YFP-tagged AhR in live Jurkat T cells. Means ± SEM are shown. (**C**) Confocal microscopy analyses of proximity ligation assays (PLA) signals from the interaction (< 40 nm) between endogenous PKCθ and AhR in two different peripheral blood T cells (#1 and #2) of Lck-GLK Tg mice. Each red dot represents for a direct interaction. Images were captured with 630× original magnification by Leica TCS SP5 Ⅱ confocal microscope. Cell nucleus was stained with DAPI. (**D**) Proximity ligation assays (PLA) of interaction between Myc-tagged PKCθ and Flag-tagged AhR in HEK293T cells. Images were captured with 400× original magnification by Leica DM2500 fluorescence microscope. Cell nucleus was stained with DAPI. (**E**) *In vitro* binding assays of purified HA-tagged AhR and GST-tagged PKCθ proteins. WT, wild-type mice; Lck-GLK, T-cell-specific GLK transgenic mice. Data shown are representatives of three independent experiments.

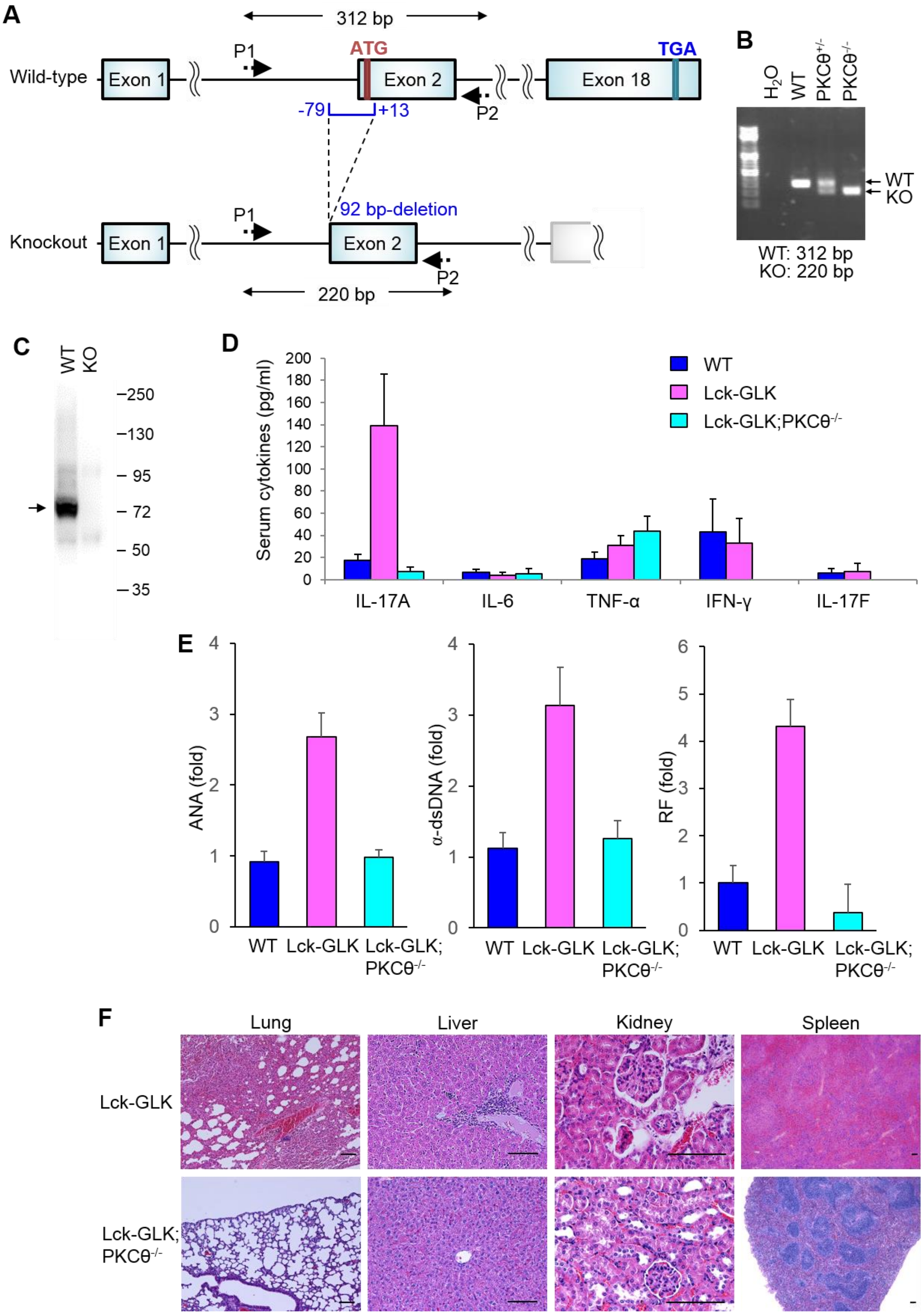
A
312 bp
Wild-type
Exon 1
P1
ATG
Exon 2
P2
TGA
Exon 18
-79
+13
92 bp-deletion
Knockout
Exon 1
P1
Exon 2
P2
220 bp
B
H2O
WT
PKCθ+/-
PKCθ-/-
WT
KO
WT: 312 bp
KO: 220 bp
C
WT
KO
250
130
95
72
50
35
D
Serum cytokines (pg/ml)
WT
Lck-GLK
Lck-GLK;PKCθ-/-
IL-17A
IL-6
TNF-α
IFN-γ
IL-17F
E
ANA (fold)
α-dsDNA (fold)
RF (fold)
WT
Lck-GLK
Lck-GLK; PKCθ-/-
F
Lung
Liver
Kidney
Spleen
Lck-GLK
Lck-GLK; PKCθ-/-

Fig. S7. Autoimmune responses in Lck-GLK Tg mice are reduced by PKCθ KO. (**A** to **C**) Generation and characterization of PKCθ knockout mice. Schematic diagram of the mouse PKCθ wild-type allele and the targeted PKCθ mutant allele (A). PKCθ knockout mice were generated by TALEN-mediated gene targeting. The deletion of 92 bp including the translation initiation codon (AUG) results in a 33 amino acid frame-shift mutant. P1 and P2, the primers for PCR. See the Methods for the details. PCR analyses of PKCθ wild-type and mutant allele using the genomic DNAs from mouse tails (B). The PCR product of the upper band (312 bp) denotes wild-type (WT) allele, and the lower band (220 bp) denotes PKCθ mutant allele. Immunoblotting of PKCθ expression in purified splenic T cells of wild-type (WT) or PKCθ knockout (KO) mice (C). (**D** to **F**) Reduction of GLK-induced autoimmune responses by PKCθ knockout. The serum levels of cytokines (D) and autoantibodies (E) in 16-week-old mice were determined by ELISA assays. n = 5 per group. Means ± SEM are shown. The autoantibody levels are presented relative to the value from one of wild-type mice. Hematoxylin and eosin-stained sections of indicated organs from 16-week-old mice (F). Bar, 100 μm. WT, wild-type littermate controls; Lck-GLK, T-cell-specific GLK transgenic mice; Lck-GLK;PKCθ$^{-/-}$, Lck-GLK transgenic mice bred with PKCθ knockout mice. ANA, anti-nuclear antibody; α-dsDNA, anti-dsDNA antibody; RF, rheumatoid factor. Data shown are representatives of three independent experiments.

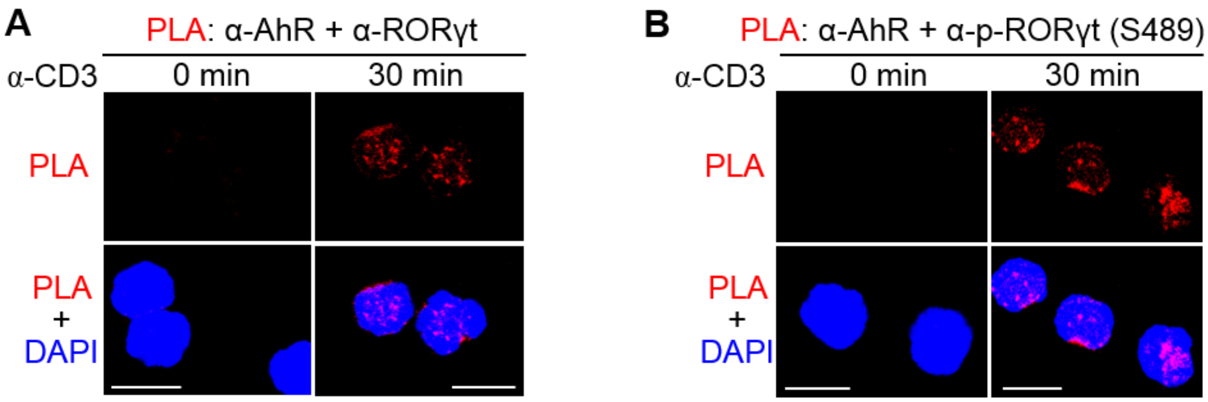

Fig. S8. TCR signaling induces in vivo interaction between AhR and RORγt. (**A** and **B**) Confocal microscopy analyses of proximity ligation assays (PLA) for the interaction between endogenous AhR and RORγt (A) or between endogenous AhR and phosphorylated RORγt (B) in murine primary T cells. T cells were stimulated with anti-CD3 antibodies plus streptavidin (3 μg each per ml). Each red dot represents for a direct interaction. T-cell nucleus was stained with DAPI (blue color). Original magnification, × 630; bar, 10 μm.

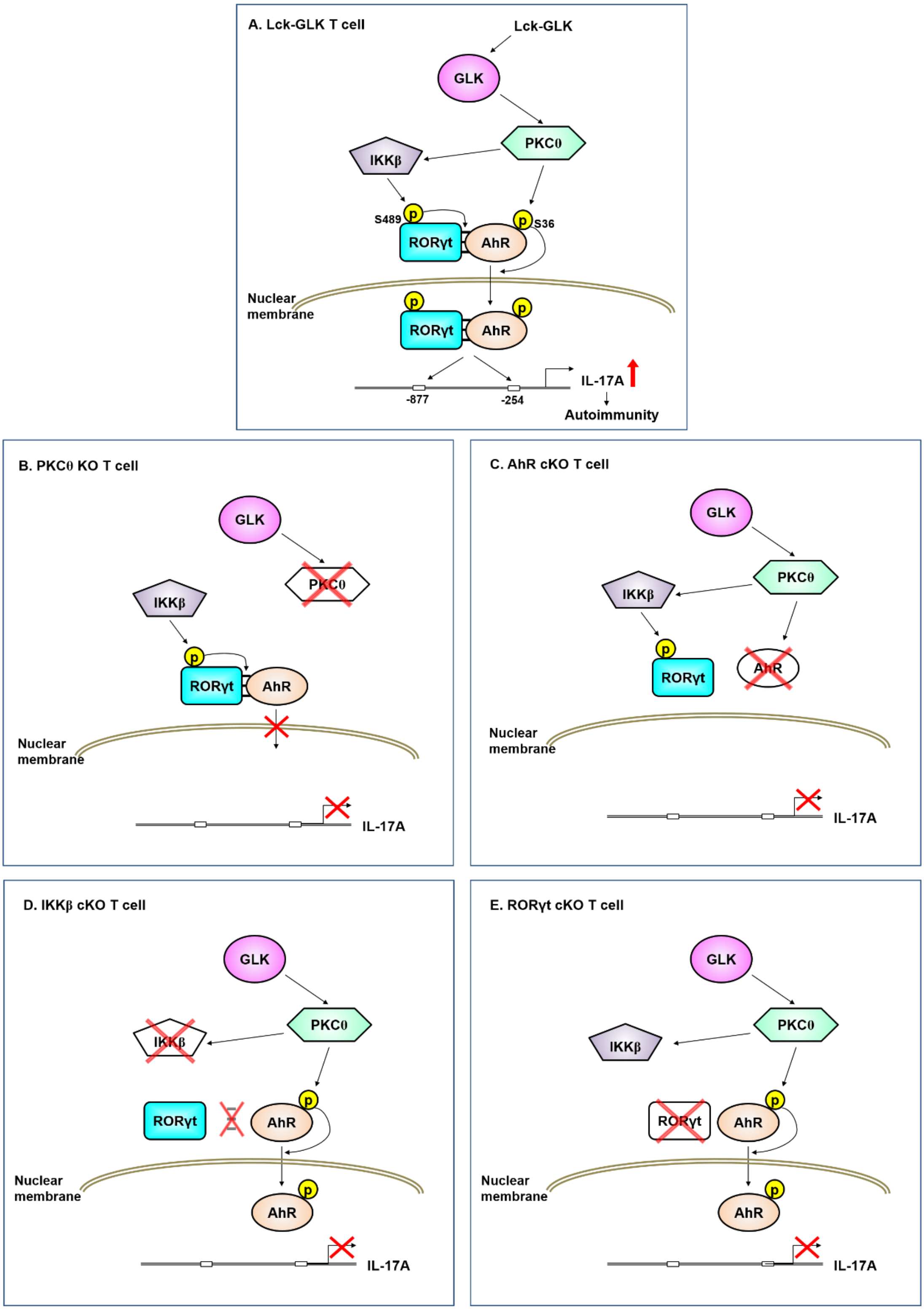

A. Lck-GLK T cell
Lck-GLK
GLK
PKCθ
IKKβ
p
S489
p
S36
RORγt
AhR
Nuclear membrane
p
p
RORγt
AhR
-877
-254
IL-17A
Autoimmunity
B. PKCθ KO T cell
GLK
PKCθ
IKKβ
p
RORγt
AhR
Nuclear membrane
IL-17A
C. AhR cKO T cell
GLK
PKCθ
IKKβ
p
RORγt
AhR
Nuclear membrane
IL-17A
D. IKKβ cKO T cell
GLK
PKCθ
IKKβ
RORγt
p
AhR
Nuclear membrane
p
AhR
IL-17A
E. RORγt cKO T cell
GLK
PKCθ
IKKβ
RORγt
p
AhR
Nuclear membrane
p
AhR
IL-17A

Fig. S9. Schematic model of AhR/RORγt-mediated IL-17A transcription in T cells of Lck-GLK Tg mice with different gene-KO backgrounds. (**A**) GLK overexpression in T cells of T-cell-specific GLK transgenic (Lck-GLK Tg) mice induces AhR Ser-36 phosphorylation through PKCθ and also induces RORγt Ser-489 phosphorylation through IKKβ. Once RORγt is phosphorylated, RORγt directly interacts with AhR. Phosphorylated AhR is responsible for transporting RORγt into cell nucleus. The RORγt-AhR complex binds to both the RORγt-binding element (-877~ -872) and AhR-binding element (-254~ -249) of the IL-17A promoter, leading to induction of IL-17A transcription and subsequent IL-17A-mediated autoimmune responses. (**B**) In T cells of Lck-GLK Tg plus AhR conditional knockout (cKO) mice, RORγt cannot be transported into the nucleus. (**C**) In T cells of Lck-GLK Tg plus PKCθ knockout mice, AhR Ser-36 is not phosphorylated and AhR localizes exclusively in the cytoplasm. Phosphorylated RORγt interacts with AhR, but cannot be transported into the nucleus. (**D**) In T cells of Lck-GLK Tg plus IKKβ conditional knockout (cKO) mice, RORγt Ser-489 is not phosphorylated. Unphosphorylated RORγt cannot interact with AhR, thus, cannot be transported into the nucleus by AhR. In contrast, only AhR translocates into the nucleus. In IKKβ cKO T cells, IL-17A induction is abolished. TCR-induced production of multiple cytokines (IL-2, IFN-γ, IL-4, IL-6, and TNF-α) are reduced in IKKβ cKO T cells due to inactivation of NF-κB. (**E**) In T cells of Lck-GLK Tg plus RORγt cKO mice, AhR is expected to translocate into the nucleus. In T cells of RORγt cKO mice, only IL-17A production is abolished, whereas NF-κB-mediated cytokines (IL-2, IFN-γ, IL-4, IL-6, and TNF-α) are not affected.

Table S1. Transcription factors of NF-κB–mediated cytokines.

**Cytokines**	**Transcription factors**	**References**
IL-2	NF-κB, NFAT1, AP-1	(*48*, *58*)
IFN-γ	NF-κB, NFAT1, AP-1, T-bet, STAT1, STAT4	(*48*, *58*, *59*)
IL-4	NF-κB, NFAT1, Egr-1, JunB, SATB1, IRF4/8	(*48*, *58*)
IL-6	NF-κB, NFAT1, IRF-1, C/EBPβ (NF-IL6)	(*48*)
TNF-α	NF-κB, NFAT1, ATF-2, Jun	(*48*)
